# Supplementary material for: Polyketide synthase gene clusters in the ericoid mycorrhizal fungus Oidiodendron maius: genome-wide prediction, expression profiling, and functional characterization of knockout mutants
Source: Front Plant Sci. 2026 Sep 16;17:1924430. doi: 10.3389/fpls.2026.1924430 (PMC13623903; doi:10.3389/fpls.2026.1924430)
Supplement: Supplementary file 1 [file SupplementaryFile1.zip › Supplementary Materials/Supplementary Folder 1.zip!/Oidma1_AssemblyScaffolds_Repeatmasked/knownclusterblast/cluster50/jgi.p_Oidma1_207831_mibig_hits.html]

| MIBiG Protein | Description | MIBiG Cluster | MiBiG Product | % ID | % Coverage | BLAST Score | E-value |
| --- | --- | --- | --- | --- | --- | --- | --- |
| AMY15068.1 | hexaketide\_synthase\_MF-SQHKS | BGC0001339 | Squalestatin\_S1\_biosynthetic\_gene\_cluster | 38 | 102.641202763 | 1565 | 0.0 |
| EPE34340.1 | Thiolase-like\_protein | BGC0001035 | Pneumocandin\_biosynthetic\_gene\_cluster | 39 | 84.7622917513 | 1309 | 0.0 |
| OAQ83760.1 | polyketide\_synthase | BGC0001358 | Leucinostatins\_biosynthetic\_gene\_cluster | 36 | 83.7058106461 | 1216 | 0.0 |
| ACB12550.1 | Fum1 | BGC0000063 | Fumonisin\_biosynthetic\_gene\_cluster | 36 | 85.0060950833 | 1206 | 0.0 |
| AAD43562.2 | Fum1p | BGC0000062 | Fumonisin\_biosynthetic\_gene\_cluster | 36 | 85.2905323039 | 1205 | 0.0 |
| BAC20566.1 | polyketide\_synthase | BGC0000039 | Compactin\_biosynthetic\_gene\_cluster | 31 | 105.526208858 | 1054 | 2e-307 |
| ABA02240.1 | polyketide\_synthase | BGC0000098 | Monacolin\_K\_biosynthetic\_gene\_cluster | 31 | 105.241771638 | 1051 | 1.3e-306 |
| AAD34559.1 | polyketide\_synthase | BGC0000089 | Lovastatin\_biosynthetic\_gene\_cluster | 31 | 105.566842747 | 1023 | 3.8e-298 |
| EHA28244.1 | hypothetical\_protein | BGC0001143 | Azanigerone\_biosynthetic\_gene\_cluster | 31 | 105.282405526 | 995 | 1.4e-289 |
| OAQ83765.1 | KR\_domain-containing\_protein | BGC0001358 | Leucinostatins\_biosynthetic\_gene\_cluster | 36 | 66.9646485169 | 940 | 5.5e-273 |
| BAD97694.1 | Aft9-1 | BGC0000003 | AF-toxin\_biosynthetic\_gene\_cluster | 27 | 101.056481105 | 777 | 3.6e-224 |
| AMY15057.1 | tetraketide\_synthase\_MF-SQTKS | BGC0001339 | Squalestatin\_S1\_biosynthetic\_gene\_cluster | 29 | 84.924827306 | 747 | 4e-215 |
| BAJ09789.1 | polyketide\_synthase | BGC0000146 | Solanapyrone\_biosynthetic\_gene\_cluster | 27 | 89.7602600569 | 720 | 5.2e-207 |
| CBF87072.1 | polyketide\_synthase,\_putative\_(Eurofung) | BGC0001290 | Emericellamide\_A\_/\_emericellamide\_B\_biosynthetic\_gene\_cluster | 33 | 65.501828525 | 695 | 2.3e-199 |
| EWG54266.1 | hypothetical\_protein | BGC0001190 | Fusaric\_acid\_biosynthetic\_gene\_cluster | 34 | 53.433563592 | 633 | 1.4e-180 |
| ACS68554.1 | hybrid\_PKS-NRPS\_protein | BGC0001026 | NG-391\_biosynthetic\_gene\_cluster | 36 | 47.2572125152 | 626 | 1e-178 |
| ACZ57548.1 | polyketide\_synthase | BGC0000046 | Depudecin\_biosynthetic\_gene\_cluster | 34 | 49.4108086144 | 626 | 1.3e-178 |
| BAV32159.1 | polyketide\_synthase | BGC0001373 | Sordarin\_biosynthetic\_gene\_cluster | 34 | 46.322633076 | 625 | 2.3e-178 |
| EHA19289.1 | hypothetical\_protein | BGC0001124 | Pyranonigrin\_E\_biosynthetic\_gene\_cluster | 34 | 51.9707436002 | 624 | 6.6e-178 |
| BAJ14522.1 | polyketide\_synthase | BGC0001253\_c4 | ACT-Toxin\_I\_biosynthetic\_gene\_cluster | 35 | 47.9073547338 | 617 | 6.2e-176 |
| AIA58899.1 | HRPKS | BGC0001141 | Brefeldin\_biosynthetic\_gene\_cluster | 34 | 51.5237708249 | 616 | 1.1e-175 |
| KGO40478.1 | Acyl\_transferase/acyl\_hydrolase/lysophospholipase | BGC0001205 | Communesin\_biosynthetic\_gene\_cluster | 35 | 50.060950833 | 613 | 1.2e-174 |
| EAL85129.1 | polyketide\_synthase,\_putative | BGC0001067 | Fumagillin\_biosynthetic\_gene\_cluster | 36 | 50.8329947176 | 612 | 2.6e-174 |
| AGC95324.1 | CurS1 | BGC0000045 | Dehydrocurvularin\_biosynthetic\_gene\_cluster | 33 | 52.3770824868 | 606 | 1.1e-172 |
| AAT28740.1 | FUSS | BGC0000064 | Fusarin\_biosynthetic\_gene\_cluster | 36 | 47.5010158472 | 601 | 4.6e-171 |
| EAW09117.1 | hybrid\_NRPS/PKS\_enzyme,\_putative | BGC0000983 | Cytochalasin\_biosynthetic\_gene\_cluster | 34 | 50.5079236083 | 601 | 4.6e-171 |
| CAO91861.1 | putative\_PKS-NRPS\_protein | BGC0000968 | Chaetoglobosin\_biosynthetic\_gene\_cluster | 36 | 47.4197480699 | 599 | 2.3e-170 |
| AFP73394.1 | FusA | BGC0001268 | Fusarin\_biosynthetic\_gene\_cluster | 36 | 47.5822836245 | 591 | 3.7e-168 |
| AHV78252.1 | ResS1 | BGC0001246 | Trans-resorcylide\_biosynthetic\_gene\_cluster | 34 | 46.444534742 | 587 | 5.3e-167 |
| AAV66110.2 | fusaridione\_A\_synthetase | BGC0000992 | Fusaridione\_A\_biosynthetic\_gene\_cluster | 35 | 48.5981308411 | 586 | 2e-166 |
| EAL85113.2 | hybrid\_PKS-NRPS\_enzyme,\_putative | BGC0001037 | Pseurotin\_A\_biosynthetic\_gene\_cluster | 34 | 48.4762291751 | 584 | 7.6e-166 |
| ACD39774.1 | reducing\_polyketide\_synthase | BGC0000134 | Radicicol\_biosynthetic\_gene\_cluster | 34 | 45.713124746 | 579 | 2.4e-164 |
| CBX87032.1 | BcBOA9\_protein | BGC0001243\_c3 | Botryenalol\_/\_botrydial\_/\_dihydrobotrydial\_/\_botryendial\_/ | 33 | 49.2889069484 | 576 | 2.1e-163 |
| AGO86662.1 | equisetin\_synthetase | BGC0001255 | Equisetin\_biosynthetic\_gene\_cluster | 36 | 47.1759447379 | 575 | 3.5e-163 |
| XP\_001220460.1 | hypothetical\_protein | BGC0001182 | Chaetoglobosins\_biosynthetic\_gene\_cluster | 30 | 65.7862657456 | 575 | 3.5e-163 |
| EAU38971.1 | hypothetical\_protein | BGC0001122 | Isoflavipucine\_biosynthetic\_gene\_cluster | 35 | 43.1125558716 | 572 | 3e-162 |
| BAD83684.1 | PKSN\_polyketide\_synthase\_for\_alternapyrone\_biosynthesis | BGC0000012 | Alternapyrone\_biosynthetic\_gene\_cluster | 33 | 49.0451036164 | 571 | 5.1e-162 |
| ACD39758.1 | reducing\_polyketide\_synthase | BGC0000076 | Hypothemycin\_biosynthetic\_gene\_cluster | 34 | 45.835026412 | 569 | 2.5e-161 |
| ACD39767.1 | reducing\_polyketide\_synthase | BGC0000077 | Hypothemycin\_biosynthetic\_gene\_cluster | 34 | 45.835026412 | 568 | 3.3e-161 |
| AKL78824.1 | GLPKS3 | BGC0001187 | Xenolozoyenone\_biosynthetic\_gene\_cluster | 32 | 54.7744819179 | 567 | 5.6e-161 |
| CBF80487.1 | hybrid\_PKS-NRPS\_(Eurofung) | BGC0000959 | Aspyridone\_biosynthetic\_gene\_cluster | 35 | 46.6477041853 | 567 | 9.6e-161 |
| ADN43685.1 | DmbS | BGC0001136 | Desmethylbassianin\_biosynthetic\_gene\_cluster | 34 | 49.4920763917 | 564 | 4.8e-160 |
| BAG17643.1 | putative\_NRPS-type-I\_PKS\_fusion\_protein | BGC0001043 | SGR\_PTMs\_biosynthetic\_gene\_cluster | 35 | 46.7289719626 | 564 | 8.2e-160 |
| AAF26922.1 | polyketide\_synthase | BGC0000988 | Epothilone\_biosynthetic\_gene\_cluster | 34 | 47.8260869565 | 561 | 4e-159 |
| CAL69597.1 | nonribosomal\_peptide\_synthase | BGC0001049 | Tenellin\_biosynthetic\_gene\_cluster | 33 | 50.304754165 | 561 | 5.3e-159 |
| CAP95405.1 |  | BGC0001404 | Sorbicillin\_biosynthetic\_gene\_cluster | 32 | 49.9796830557 | 560 | 9e-159 |
| AAF62884.1 | EpoE | BGC0000991 | Epothilone\_biosynthetic\_gene\_cluster | 34 | 47.6229175132 | 559 | 1.5e-158 |
| ADB12492.1 | EpoE | BGC0000990 | Epothilone\_biosynthetic\_gene\_cluster | 34 | 48.4355952865 | 558 | 3.4e-158 |
| ACB46196.1 | polyketide\_synthase | BGC0000989 | Epothilone\_biosynthetic\_gene\_cluster | 34 | 46.9321414059 | 557 | 5.8e-158 |
| AAK57188.1 | MxaD | BGC0001022 | Myxalamid\_biosynthetic\_gene\_cluster | 33 | 45.9975619667 | 555 | 2.9e-157 |
| AKC54422.1 | fumosorinone\_biosynthesis\_polyketide\_synthase | BGC0001218 | Fumosorinone\_biosynthetic\_gene\_cluster | 34 | 50.060950833 | 555 | 2.9e-157 |
| AHV78245.1 | LasS1 | BGC0001245 | Lasiodiplodin\_biosynthetic\_gene\_cluster | 33 | 46.322633076 | 554 | 6.5e-157 |
| CAG28678.1 | polyketide\_synthase | BGC0001023 | Myxochromide\_biosynthetic\_gene\_cluster | 34 | 46.8102397399 | 552 | 3.2e-156 |
| AGC45621.1 | polyketide\_synthase | BGC0001394 | Phenalamide\_biosynthetic\_gene\_cluster | 34 | 46.322633076 | 551 | 5.5e-156 |
| ABA02239.1 | polyketide\_synthase | BGC0000098 | Monacolin\_K\_biosynthetic\_gene\_cluster | 34 | 48.5168630638 | 550 | 1.2e-155 |
| EFL02193.1 | amino\_acid\_adenylation\_domain-containing\_protein | BGC0000996 | Frontalamides\_biosynthetic\_gene\_cluster | 35 | 46.6070702966 | 549 | 2.1e-155 |
| ABB90283.1 | polyketide\_synthase | BGC0001057 | Zearalenone\_biosynthetic\_gene\_cluster | 33 | 45.3067858594 | 547 | 6e-155 |
| ctg1\_orf0002 |  | BGC0001068 | Pyripyropene\_A\_biosynthetic\_gene\_cluster | 29 | 67.6960585128 | 544 | 8.7e-154 |
| BAQ25466.1 | polyketide\_synthase | BGC0001280 | Betaenone\_C\_/\_betaenone\_A\_biosynthetic\_gene\_cluster | 36 | 41.731003657 | 544 | 8.7e-154 |
| CCE88376.1 | polyketide\_synthase | BGC0001034 | Pellasoren\_biosynthetic\_gene\_cluster | 33 | 47.0946769606 | 542 | 3.3e-153 |
| BAK26562.1 | PKS-NRPS\_hybrid | BGC0000977 | Cyclopiazonic\_acid\_biosynthetic\_gene\_cluster | 33 | 48.3949613978 | 540 | 9.7e-153 |
| AAF26921.1 | polyketide\_synthase | BGC0000988 | Epothilone\_biosynthetic\_gene\_cluster | 33 | 46.8102397399 | 540 | 1.3e-152 |
| EAL89230.2 | LovB-like\_polyketide\_synthase,\_putative | BGC0000129 | Pyripyropene\_biosynthetic\_gene\_cluster | 35 | 41.9341731004 | 538 | 3.7e-152 |
| ACB46195.1 | polyketide\_synthase | BGC0000989 | Epothilone\_biosynthetic\_gene\_cluster | 33 | 46.8102397399 | 538 | 3.7e-152 |
| BAC20564.1 | polyketide\_synthase | BGC0000039 | Compactin\_biosynthetic\_gene\_cluster | 32 | 49.695245835 | 538 | 4.8e-152 |
| ADB12491.1 | EpoD | BGC0000990 | Epothilone\_biosynthetic\_gene\_cluster | 33 | 46.8102397399 | 537 | 8.2e-152 |
| AAF62883.1 | epoD | BGC0000991 | Epothilone\_biosynthetic\_gene\_cluster | 33 | 46.8102397399 | 537 | 8.2e-152 |
| AAF19810.1 | MtaB | BGC0001024 | Myxothiazol\_biosynthetic\_gene\_cluster | 34 | 46.8102397399 | 537 | 8.2e-152 |
| EAA65604.1 | hypothetical\_protein | BGC0000022 | Asperfuranone\_biosynthetic\_gene\_cluster | 32 | 47.5010158472 | 530 | 1.3e-149 |
| CCT72377.1 | probable\_polyketide\_synthase | BGC0001305 | Fujikurins\_biosynthetic\_gene\_cluster | 32 | 51.9301097115 | 529 | 2.9e-149 |
| ABL86391.1 | hybrid\_polyketide\_synthase\_and\_nonribosomal\_peptide\_synthetase | BGC0000999 | Heat-stable\_antifungal\_factor\_biosynthetic\_gene\_cluster | 33 | 46.444534742 | 527 | 8.5e-149 |
| CAQ43078.1 | polyketide\_synthase | BGC0000970 | Chondrochloren\_biosynthetic\_gene\_cluster | 32 | 49.939049167 | 526 | 1.4e-148 |
| AGC45624.1 | polyketide\_synthase | BGC0001394 | Phenalamide\_biosynthetic\_gene\_cluster | 33 | 46.2413652987 | 522 | 2.7e-147 |
| AGC45619.1 | polyketide\_synthase | BGC0001394 | Phenalamide\_biosynthetic\_gene\_cluster | 32 | 49.7358797237 | 521 | 6.1e-147 |
| ALD82522.1 | polyketide\_synthase | BGC0001212 | Nannocystin\_biosynthetic\_gene\_cluster | 33 | 46.8508736286 | 520 | 1e-146 |
| ANI24099.1 | polyketide\_synthase | BGC0001235 | Antalid\_biosynthetic\_gene\_cluster | 32 | 50.9955302722 | 520 | 1.4e-146 |
| CCE88379.1 | polyketide\_synthase | BGC0001034 | Pellasoren\_biosynthetic\_gene\_cluster | 32 | 49.573344169 | 519 | 3e-146 |
| CAQ43076.1 | polyketide\_synthase | BGC0000970 | Chondrochloren\_biosynthetic\_gene\_cluster | 31 | 52.1332791548 | 517 | 6.7e-146 |
| CCE88380.1 | polyketide\_synthase | BGC0001034 | Pellasoren\_biosynthetic\_gene\_cluster | 32 | 50.5079236083 | 517 | 8.8e-146 |
| CCE88378.1 | polyketide\_synthase | BGC0001034 | Pellasoren\_biosynthetic\_gene\_cluster | 31 | 50.3860219423 | 515 | 4.3e-145 |
| CAQ18830.1 | polyketide\_synthase | BGC0000954 | Ajudazol\_biosynthetic\_gene\_cluster | 32 | 47.1353108492 | 514 | 5.7e-145 |
| EFY94490.1 | polyketide\_synthase,\_putative | BGC0000337 | Destruxins\_biosynthetic\_gene\_cluster | 34 | 40.6745225518 | 512 | 2.8e-144 |
| CCE88381.1 | polyketide\_synthase | BGC0001034 | Pellasoren\_biosynthetic\_gene\_cluster | 31 | 49.5327102804 | 511 | 4.8e-144 |
| AAK57189.1 | MxaE | BGC0001022 | Myxalamid\_biosynthetic\_gene\_cluster | 33 | 47.5416497359 | 510 | 8.2e-144 |
| AIW82279.1 | PuwB | BGC0001125 | Puwainaphycins\_biosynthetic\_gene\_cluster | 32 | 46.688338074 | 509 | 2.4e-143 |
| AGC45622.1 | polyketide\_synthase | BGC0001394 | Phenalamide\_biosynthetic\_gene\_cluster | 32 | 46.078829744 | 504 | 7.7e-142 |
| AGC45620.1 | polyketide\_synthase | BGC0001394 | Phenalamide\_biosynthetic\_gene\_cluster | 36 | 36.9362047948 | 499 | 1.9e-140 |
| AAF19813.1 | MtaE | BGC0001024 | Myxothiazol\_biosynthetic\_gene\_cluster | 36 | 37.0174725721 | 499 | 2.5e-140 |
| ACR33077.1 | polyketide\_synthase | BGC0000017 | Anatoxin\_biosynthetic\_gene\_cluster | 31 | 46.6477041853 | 498 | 5.5e-140 |
| BAF02926.1 | type\_I\_polyketide\_synthase | BGC0000073 | Halstoctacosanolide\_biosynthetic\_gene\_cluster | 31 | 46.566436408 | 495 | 4.7e-139 |
| AGC45623.1 | polyketide\_synthase | BGC0001394 | Phenalamide\_biosynthetic\_gene\_cluster | 32 | 46.20073141 | 493 | 1.4e-138 |
| AAW03328.1 | CtaE | BGC0000982 | Cystothiazole\_A\_biosynthetic\_gene\_cluster | 36 | 36.8955709061 | 490 | 8.8e-138 |
| ABM21570.1 | CrpB | BGC0000975 | Cryptophycin\_biosynthetic\_gene\_cluster | 30 | 49.451442503 | 490 | 1.5e-137 |
| CAJ46690.1 | polyketide\_synthase | BGC0000969 | Chondramide\_biosynthetic\_gene\_cluster | 32 | 46.566436408 | 490 | 1.5e-137 |
| AAS98783.1 | JamL | BGC0001001 | Jamaicamide\_biosynthetic\_gene\_cluster | 30 | 47.9886225112 | 489 | 2e-137 |
| BAN19720.1 | polyketide\_synthase | BGC0001252 | ACR-toxin\_biosynthetic\_gene\_cluster | 32 | 46.1194636327 | 489 | 2.6e-137 |
| AEU17899.1 | putative\_type\_I\_PKS | BGC0001072 | Simocyclinone\_biosynthetic\_gene\_cluster | 31 | 46.3632669646 | 489 | 2.6e-137 |
| EAQ86385.1 | hypothetical\_protein | BGC0001405 | Chaetoviridin\_/\_chaetomugilin\_biosynthetic\_gene\_cluster | 36 | 36.8143031288 | 488 | 3.3e-137 |
| CAQ43075.1 | polyketide\_synthase | BGC0000970 | Chondrochloren\_biosynthetic\_gene\_cluster | 31 | 47.0134091833 | 488 | 3.3e-137 |
| ABB76806.1 | polyketide\_synthase\_2 | BGC0000155\_c2 | T-toxin\_biosynthetic\_gene\_cluster | 32 | 47.1759447379 | 487 | 9.7e-137 |
| ADM46358.1 | polyketide\_synthase | BGC0000106 | Naphthomycin\_biosynthetic\_gene\_cluster | 32 | 46.2819991873 | 487 | 1.3e-136 |
| AIT55259.1 | GulB | BGC0000072 | Gulmirecin\_biosynthetic\_gene\_cluster | 36 | 37.0174725721 | 486 | 2.2e-136 |
| CAD19091.1 | StiG\_protein | BGC0000153 | Stigmatellin\_biosynthetic\_gene\_cluster | 31 | 46.7696058513 | 485 | 2.8e-136 |
| ACR33078.1 | polyketide\_synthase | BGC0000017 | Anatoxin\_biosynthetic\_gene\_cluster | 29 | 49.6139780577 | 482 | 2.4e-135 |
| ABV97152.1 | Beta-ketoacyl\_synthase | BGC0000137 | Rifamycin\_biosynthetic\_gene\_cluster | 31 | 45.59122308 | 482 | 2.4e-135 |
| AIT55260.1 | GulC | BGC0000072 | Gulmirecin\_biosynthetic\_gene\_cluster | 31 | 50.7110930516 | 482 | 4.1e-135 |
| CBD77734.1 | polyketide\_synthase | BGC0000974 | Crocacin\_biosynthetic\_gene\_cluster | 35 | 36.5704997968 | 481 | 7e-135 |
| AHN85651.1 | Phn2 | BGC0000122 | Phenylnannolone\_A\_biosynthetic\_gene\_cluster | 31 | 46.9321414059 | 478 | 3.5e-134 |
| CAL58681.1 | polyketide\_synthase | BGC0000149 | Spirangien\_biosynthetic\_gene\_cluster | 30 | 52.2958147095 | 478 | 4.5e-134 |
| ABK32255.1 | AmbA | BGC0000014 | Ambruticin\_biosynthetic\_gene\_cluster | 35 | 37.0581064608 | 478 | 5.9e-134 |
| CAQ18829.1 | polyketide\_synthase | BGC0000954 | Ajudazol\_biosynthetic\_gene\_cluster | 31 | 47.2572125152 | 476 | 1.7e-133 |
| BAF85839.1 | modular\_polyketide\_synthase | BGC0000109 | Nemadectin\_biosynthetic\_gene\_cluster | 31 | 47.1353108492 | 475 | 2.9e-133 |
| ADH04660.1 | TugD | BGC0001342 | Thuggacin\_biosynthetic\_gene\_cluster | 31 | 46.566436408 | 475 | 3.8e-133 |
| CBD77736.1 | polyketide\_synthase | BGC0000974 | Crocacin\_biosynthetic\_gene\_cluster | 32 | 46.8508736286 | 475 | 5e-133 |
| AAK57187.1 | MxaC | BGC0001022 | Myxalamid\_biosynthetic\_gene\_cluster | 35 | 36.6517675742 | 474 | 6.5e-133 |
| AAF26920.1 | polyketide\_synthase | BGC0000988 | Epothilone\_biosynthetic\_gene\_cluster | 32 | 46.8915075173 | 474 | 6.5e-133 |
| CAD19089.1 | StiE\_protein | BGC0000153 | Stigmatellin\_biosynthetic\_gene\_cluster | 34 | 36.5704997968 | 474 | 8.5e-133 |
| AJW65409.1 | type\_I\_modular\_polyketide\_synthase | BGC0001195 | Nocardiopsin\_biosynthetic\_gene\_cluster | 31 | 46.566436408 | 473 | 1.1e-132 |
| AEE88279.1 | CurK | BGC0000976 | Curacin\_biosynthetic\_gene\_cluster | 29 | 47.6635514019 | 472 | 2.5e-132 |
| CQR60496.1 | Polyketide\_synthase,\_type\_I,\_modules:\_4,\_5\_and\_6 | BGC0001287 | Chaxamycin\_A\_/\_chaxamycin\_B\_/\_chaxamycin\_C\_/\_chaxamycin\_D | 31 | 45.956928078 | 472 | 2.5e-132 |
| AAT70106.1 | CurK | BGC0001165 | Curacin\_biosynthetic\_gene\_cluster | 29 | 47.6635514019 | 472 | 2.5e-132 |
| AKD43522.1 | Type\_I\_polyketide\_synthase | BGC0001409 | Dutomycin\_biosynthetic\_gene\_cluster | 30 | 52.9865908167 | 472 | 3.2e-132 |
| BAQ25513.1 | type\_I\_polyketide\_synthase | BGC0001288 | Maklamicin\_biosynthetic\_gene\_cluster | 32 | 46.0381958553 | 472 | 3.2e-132 |
| ABK32287.1 | JerA | BGC0000080 | Jerangolid\_biosynthetic\_gene\_cluster | 35 | 36.8549370175 | 472 | 3.2e-132 |
| CAL58684.1 | polyketide\_synthase | BGC0000149 | Spirangien\_biosynthetic\_gene\_cluster | 31 | 47.2978464039 | 471 | 5.5e-132 |
| ADZ24997.1 | polyketide\_synthase | BGC0000380 | Leupyrrin\_biosynthetic\_gene\_cluster | 31 | 46.7289719626 | 471 | 5.5e-132 |
| ACY13414.1 | amino\_acid\_adenylation\_domain\_protein | BGC0001367 | Haliamide\_biosynthetic\_gene\_cluster | 31 | 46.3632669646 | 471 | 7.2e-132 |
| CAD89776.1 | MelE\_protein | BGC0001010 | Melithiazol\_biosynthetic\_gene\_cluster | 35 | 36.8955709061 | 470 | 9.4e-132 |
| AAK19883.1 | soraphen\_polyketide\_synthase\_A | BGC0000147 | Soraphen\_biosynthetic\_gene\_cluster | 30 | 46.4039008533 | 470 | 1.2e-131 |
| AEE88280.1 | CurJ | BGC0000976 | Curacin\_biosynthetic\_gene\_cluster | 30 | 48.5574969525 | 469 | 2.1e-131 |
| AAT70105.1 | CurJ | BGC0001165 | Curacin\_biosynthetic\_gene\_cluster | 30 | 48.5574969525 | 469 | 2.1e-131 |
| AJW65407.1 | type\_I\_modular\_polyketide\_synthase | BGC0001195 | Nocardiopsin\_biosynthetic\_gene\_cluster | 32 | 46.8102397399 | 469 | 2.1e-131 |
| CAM00065.1 | EryAIII\_Erythromycin\_polyketide\_synthase\_modules\_5\_and\_6 | BGC0000055 | Erythromycin\_biosynthetic\_gene\_cluster | 33 | 37.2612759041 | 468 | 3.6e-131 |
| ACB46194.1 | polyketide\_synthase | BGC0000989 | Epothilone\_biosynthetic\_gene\_cluster | 31 | 46.4851686306 | 468 | 3.6e-131 |
| AAF62882.1 | EpoC | BGC0000991 | Epothilone\_biosynthetic\_gene\_cluster | 31 | 46.4851686306 | 468 | 3.6e-131 |
| ADB12490.1 | EpoC | BGC0000990 | Epothilone\_biosynthetic\_gene\_cluster | 31 | 46.6070702966 | 468 | 3.6e-131 |
| ADH04639.1 | TgaA | BGC0001051 | Thuggacin\_biosynthetic\_gene\_cluster | 30 | 50.6298252743 | 468 | 4.7e-131 |
| CBD77738.1 | polyketide\_synthase | BGC0000974 | Crocacin\_biosynthetic\_gene\_cluster | 35 | 37.1393742381 | 468 | 4.7e-131 |
| WP\_042799407.1 | polyketide\_synthase | BGC0001283 | Arsenopolyketides\_biosynthetic\_gene\_cluster | 31 | 46.7696058513 | 468 | 6.1e-131 |
| CAD19090.1 | StiF\_protein | BGC0000153 | Stigmatellin\_biosynthetic\_gene\_cluster | 30 | 46.444534742 | 468 | 6.1e-131 |
| AIT55261.1 | GulD | BGC0000072 | Gulmirecin\_biosynthetic\_gene\_cluster | 36 | 36.7736692401 | 467 | 8e-131 |
| AAC01712.2 | RifC | BGC0000136 | Rifamycin\_biosynthetic\_gene\_cluster | 32 | 45.7537586347 | 467 | 1e-130 |
| AAX98191.1 | polyketide\_synthase\_type\_I | BGC0000052 | ECO-02301\_biosynthetic\_gene\_cluster | 31 | 46.0381958553 | 467 | 1.4e-130 |
| BAH02268.1 | polyketide\_synthase | BGC0000126 | Pladienolide\_biosynthetic\_gene\_cluster | 30 | 46.9321414059 | 466 | 2.3e-130 |
| AAC38075.1 | polyketide\_synthase\_type\_I | BGC0000127 | Pyoluteorin\_biosynthetic\_gene\_cluster | 34 | 36.5298659082 | 465 | 3.9e-130 |
| ABV97153.1 | Beta-ketoacyl\_synthase | BGC0000137 | Rifamycin\_biosynthetic\_gene\_cluster | 31 | 45.956928078 | 465 | 3.9e-130 |
| ABB88533.1 | polyketide\_synthase\_type\_I | BGC0000049 | E-492\_/\_E-975\_biosynthetic\_gene\_cluster | 29 | 51.3612352702 | 465 | 5.2e-130 |
| ADH04641.1 | TgaC | BGC0001051 | Thuggacin\_biosynthetic\_gene\_cluster | 30 | 46.2819991873 | 465 | 5.2e-130 |
| ADC79618.1 | BafAIII | BGC0000028 | Bafilomycin\_biosynthetic\_gene\_cluster | 31 | 46.688338074 | 464 | 8.8e-130 |
| ABY83164.1 | Azi26 | BGC0000960 | Azinomycin\_B\_biosynthetic\_gene\_cluster | 31 | 42.218610321 | 464 | 8.8e-130 |
| ADC45538.1 | modular\_polyketide\_synthase | BGC0000093 | Meilingmycin\_biosynthetic\_gene\_cluster | 29 | 47.4603819586 | 464 | 8.8e-130 |
| AAC01711.1 | RifB | BGC0000136 | Rifamycin\_biosynthetic\_gene\_cluster | 30 | 51.2799674929 | 464 | 8.8e-130 |
| AAF86393.1 | FkbB | BGC0000994 | FK520\_biosynthetic\_gene\_cluster | 31 | 45.835026412 | 463 | 1.1e-129 |
| CBD77732.1 | polyketide\_synthase | BGC0000974 | Crocacin\_biosynthetic\_gene\_cluster | 34 | 37.1800081268 | 463 | 1.5e-129 |
| ADX66459.1 | ScnS4 | BGC0000108 | Natamycin\_biosynthetic\_gene\_cluster | 31 | 45.4693214141 | 463 | 2e-129 |
| CAD19085.1 | StiA\_protein | BGC0000153 | Stigmatellin\_biosynthetic\_gene\_cluster | 36 | 35.5546525803 | 463 | 2e-129 |
| CQR60495.1 | Polyketide\_synthase,\_type\_I,\_module\_7 | BGC0001287 | Chaxamycin\_A\_/\_chaxamycin\_B\_/\_chaxamycin\_C\_/\_chaxamycin\_D | 31 | 45.835026412 | 463 | 2e-129 |
| CAO85896.1 | protein\_modular\_polyketide\_synthase\_NorA' | BGC0000110 | Neoaureothin\_biosynthetic\_gene\_cluster | 31 | 46.20073141 | 462 | 2.6e-129 |
| ABK32259.1 | AmbE | BGC0000014 | Ambruticin\_biosynthetic\_gene\_cluster | 30 | 46.3632669646 | 462 | 2.6e-129 |
| BAF85843.1 | modular\_polyketide\_synthase | BGC0000109 | Nemadectin\_biosynthetic\_gene\_cluster | 30 | 46.7289719626 | 462 | 2.6e-129 |
| AEP40934.1 | polyketide\_synthase\_type\_I | BGC0000021 | Apoptolidin\_biosynthetic\_gene\_cluster | 29 | 48.6793986184 | 462 | 3.3e-129 |
| CAQ18835.1 | polyketide\_synthase | BGC0000954 | Ajudazol\_biosynthetic\_gene\_cluster | 32 | 43.4782608696 | 462 | 4.4e-129 |
| AFI57006.1 | QmnA2 | BGC0000133 | Quartromicin\_biosynthetic\_gene\_cluster | 31 | 46.6070702966 | 461 | 5.7e-129 |
| AGY30676.1 | Ann4 | BGC0001298 | 4-Z-annimycin\_biosynthetic\_gene\_cluster | 30 | 46.6477041853 | 461 | 5.7e-129 |
| CBD77748.1 | polyketide\_synthase | BGC0000974 | Crocacin\_biosynthetic\_gene\_cluster | 31 | 46.8915075173 | 461 | 7.4e-129 |
| ADU86002.1 | putative\_modular\_polyketide\_synthase | BGC0000165 | Tiacumicin\_B\_biosynthetic\_gene\_cluster | 31 | 45.6318569687 | 461 | 7.4e-129 |
| AEZ54374.1 | PieA1 | BGC0000124 | Piericidin\_A1\_biosynthetic\_gene\_cluster | 31 | 46.1600975213 | 460 | 9.7e-129 |
| ABK32257.1 | AmbC | BGC0000014 | Ambruticin\_biosynthetic\_gene\_cluster | 31 | 46.7289719626 | 460 | 1.7e-128 |
| CAL58682.1 | polyketide\_synthase | BGC0000149 | Spirangien\_biosynthetic\_gene\_cluster | 30 | 46.6070702966 | 460 | 1.7e-128 |
| ADC45515.1 | modular\_polyketide\_synthase | BGC0000093 | Meilingmycin\_biosynthetic\_gene\_cluster | 31 | 46.3632669646 | 459 | 2.2e-128 |
| CAQ18832.1 | polyketide\_synthase | BGC0000954 | Ajudazol\_biosynthetic\_gene\_cluster | 31 | 47.0134091833 | 458 | 3.7e-128 |
| CAE45671.1 | borrelidin\_polyketide\_synthase,\_type\_I | BGC0000031 | Borrelidin\_biosynthetic\_gene\_cluster | 31 | 47.0946769606 | 458 | 4.8e-128 |
| CAQ34928.1 | polyketide\_synthase | BGC0000986 | Dkxanthene\_biosynthetic\_gene\_cluster | 32 | 46.8102397399 | 457 | 8.2e-128 |
| AEZ64505.1 | Herb | BGC0001065 | Herboxidiene\_biosynthetic\_gene\_cluster | 31 | 46.566436408 | 457 | 8.2e-128 |
| AFI57005.1 | QmnA1 | BGC0000133 | Quartromicin\_biosynthetic\_gene\_cluster | 31 | 45.6318569687 | 457 | 1.1e-127 |
| CQR60494.1 | Polyketide\_synthase,\_type\_I,\_module\_8 | BGC0001287 | Chaxamycin\_A\_/\_chaxamycin\_B\_/\_chaxamycin\_C\_/\_chaxamycin\_D | 32 | 42.5030475416 | 457 | 1.1e-127 |
| CAO98850.1 | polyketide\_synthase\_AufG | BGC0000023 | Aurafuron\_biosynthetic\_gene\_cluster | 30 | 46.3632669646 | 456 | 1.4e-127 |
| AAA79984.2 | soraphen\_polyketide\_synthase\_B | BGC0000147 | Soraphen\_biosynthetic\_gene\_cluster | 30 | 46.566436408 | 456 | 1.8e-127 |
| AGY30677.1 | Ann5 | BGC0001298 | 4-Z-annimycin\_biosynthetic\_gene\_cluster | 31 | 46.688338074 | 456 | 1.8e-127 |
| ACB37755.1 | putative\_type\_I\_polyketide\_synthase | BGC0000162 | Tetrocarcin\_A\_biosynthetic\_gene\_cluster | 31 | 47.0540430719 | 456 | 1.8e-127 |
| ABJ97438.1 | MerB | BGC0001012 | Meridamycin\_biosynthetic\_gene\_cluster | 30 | 47.2165786266 | 456 | 1.8e-127 |
| CAC20919.1 | PimS4\_protein | BGC0000125 | Pimaricin\_biosynthetic\_gene\_cluster | 31 | 45.5505891914 | 456 | 1.8e-127 |
| AAC01710.1 | RifA | BGC0000136 | Rifamycin\_biosynthetic\_gene\_cluster | 35 | 36.4892320195 | 456 | 1.8e-127 |
| AAG13917.1 | megalomicin\_6-deoxyerythronolide\_B\_synthase\_1 | BGC0000092 | Megalomicin\_biosynthetic\_gene\_cluster | 35 | 36.7736692401 | 455 | 3.1e-127 |
| ADC45535.1 | modular\_polyketide\_synthase | BGC0000093 | Meilingmycin\_biosynthetic\_gene\_cluster | 33 | 36.7330353515 | 455 | 5.3e-127 |
| BAJ16467.1 | polyketide\_synthase | BGC0000058 | FD-891\_biosynthetic\_gene\_cluster | 30 | 54.043071922 | 454 | 7e-127 |
| ABC87510.1 | polyketide\_synthase | BGC0001011 | Meridamycin\_biosynthetic\_gene\_cluster | 30 | 47.2572125152 | 454 | 7e-127 |
| ctg1\_orf21 |  | BGC0001013 | Meridamycin\_biosynthetic\_gene\_cluster | 30 | 47.2572125152 | 454 | 7e-127 |
| CAQ34920.1 | polyketide\_synthase | BGC0000986 | Dkxanthene\_biosynthetic\_gene\_cluster | 30 | 49.2889069484 | 454 | 7e-127 |
| ctg1\_orf29 |  | BGC0000096 | Midecamycin\_biosynthetic\_gene\_cluster | 31 | 46.5258025193 | 454 | 9.1e-127 |
| ACY06289.1 | type\_I\_polyketide\_synthase | BGC0001042 | Sanglifehrin\_A\_biosynthetic\_gene\_cluster | 31 | 46.1600975213 | 453 | 1.2e-126 |
| ACO94460.1 | polyketide\_synthase\_type\_I | BGC0000029 | BE-14106\_biosynthetic\_gene\_cluster | 30 | 46.0381958553 | 453 | 1.2e-126 |
| BAO66542.1 | type\_I\_polyketide\_synthase | BGC0000042 | Cremimycin\_biosynthetic\_gene\_cluster | 30 | 46.7289719626 | 453 | 1.2e-126 |
| BAB69199.1 | modular\_polyketide\_synthase | BGC0000117 | Oligomycin\_biosynthetic\_gene\_cluster | 31 | 46.8508736286 | 453 | 1.2e-126 |
| ACN64831.1 | PokM1 | BGC0001061 | Polyketomycin\_biosynthetic\_gene\_cluster | 33 | 40.1869158879 | 453 | 1.2e-126 |
| ACY06286.1 | polyketide\_synthase | BGC0001042 | Sanglifehrin\_A\_biosynthetic\_gene\_cluster | 31 | 45.713124746 | 453 | 1.2e-126 |
| ABB05103.1 | LipPks2 | BGC0001003 | Lipomycin\_biosynthetic\_gene\_cluster | 30 | 46.8102397399 | 453 | 1.6e-126 |
| CCP20047.1 | divK\_protein | BGC0001119 | Divergolide\_biosynthetic\_gene\_cluster | 34 | 36.8955709061 | 453 | 1.6e-126 |
| BAO66529.1 | type\_I\_polyketide\_synthase | BGC0000042 | Cremimycin\_biosynthetic\_gene\_cluster | 30 | 50.9142624949 | 453 | 2e-126 |
| ADH04657.1 | TugA | BGC0001342 | Thuggacin\_biosynthetic\_gene\_cluster | 30 | 46.7696058513 | 453 | 2e-126 |
| AAZ77696.1 | ChlA3 | BGC0000036 | Chlorothricin\_biosynthetic\_gene\_cluster | 31 | 46.5258025193 | 453 | 2e-126 |
| ABK32290.1 | JerD | BGC0000080 | Jerangolid\_biosynthetic\_gene\_cluster | 30 | 49.9796830557 | 453 | 2e-126 |
| BAE93729.1 | type\_I\_polyketide\_synthase | BGC0000164 | Tetronomycin\_biosynthetic\_gene\_cluster | 30 | 47.2165786266 | 452 | 2.6e-126 |
| CAO98852.1 | polyketide\_synthase\_AufI | BGC0000023 | Aurafuron\_biosynthetic\_gene\_cluster | 35 | 36.8143031288 | 452 | 2.6e-126 |
| CAO98849.1 | polyketide\_synthase\_AufF | BGC0000023 | Aurafuron\_biosynthetic\_gene\_cluster | 33 | 36.9362047948 | 452 | 2.6e-126 |
| ADM46360.1 | polyketide\_synthase | BGC0000106 | Naphthomycin\_biosynthetic\_gene\_cluster | 31 | 47.5010158472 | 452 | 3.5e-126 |
| ABX60163.1 | polyketide\_synthase | BGC0000978 | Cylindrospermopsin\_biosynthetic\_gene\_cluster | 33 | 39.6586753352 | 451 | 4.5e-126 |
| ALA09371.1 | type\_I\_modular\_PKS | BGC0001303 | Lobosamide\_biosynthetic\_gene\_cluster | 30 | 46.4851686306 | 451 | 4.5e-126 |
| ADM46357.1 | polyketide\_synthase | BGC0000106 | Naphthomycin\_biosynthetic\_gene\_cluster | 31 | 46.1194636327 | 451 | 7.7e-126 |
| AEE88282.1 | CurH | BGC0000976 | Curacin\_biosynthetic\_gene\_cluster | 31 | 46.4039008533 | 451 | 7.7e-126 |
| ABV97155.1 | Acyl\_transferase | BGC0000137 | Rifamycin\_biosynthetic\_gene\_cluster | 30 | 46.078829744 | 451 | 7.7e-126 |
| AAT70103.1 | CurH | BGC0001165 | Curacin\_biosynthetic\_gene\_cluster | 31 | 46.4039008533 | 451 | 7.7e-126 |
| CAQ34919.1 | polyketide\_synthase | BGC0000986 | Dkxanthene\_biosynthetic\_gene\_cluster | 30 | 46.4851686306 | 450 | 1e-125 |
| BAC76492.1 | lankamycin\_synthase\_LkmAII | BGC0000085 | Lankamycin\_biosynthetic\_gene\_cluster | 34 | 36.4485981308 | 450 | 1e-125 |
| BAQ21947.1 | putative\_type\_I\_polyketide\_synthase | BGC0001204 | Versipelostatin\_biosynthetic\_gene\_cluster | 30 | 47.4197480699 | 450 | 1.3e-125 |
| ABW96542.1 | type\_I\_modular\_polyketide\_synthase | BGC0000159 | Tautomycin\_biosynthetic\_gene\_cluster | 29 | 48.2730597318 | 450 | 1.3e-125 |
| ACO94488.1 | polyketide\_synthase\_type\_I | BGC0000097 | ML-449\_biosynthetic\_gene\_cluster | 30 | 46.688338074 | 450 | 1.7e-125 |
| ADF88277.1 | polyketide\_synthase | BGC0000981 | Cylindrospermopsin\_biosynthetic\_gene\_cluster | 32 | 39.6586753352 | 450 | 1.7e-125 |
| CAO85893.1 | modular\_polyketide\_synthase\_NorA | BGC0000110 | Neoaureothin\_biosynthetic\_gene\_cluster | 30 | 46.4851686306 | 450 | 1.7e-125 |
| CAE02602.1 | polyketide\_synthase\_type\_I | BGC0000024 | Aureothin\_biosynthetic\_gene\_cluster | 30 | 46.688338074 | 449 | 2.2e-125 |
| AIT55264.1 | GulA | BGC0000072 | Gulmirecin\_biosynthetic\_gene\_cluster | 35 | 36.1641609102 | 449 | 2.2e-125 |
| CAI94713.1 | putative\_polyketide\_synthase | BGC0000141 | Rubradirin\_biosynthetic\_gene\_cluster | 30 | 45.9162941894 | 449 | 2.9e-125 |
| AAC01713.1 | RifD | BGC0000136 | Rifamycin\_biosynthetic\_gene\_cluster | 31 | 45.713124746 | 449 | 2.9e-125 |
| AAK83194.1 | polyketide\_synthase | BGC0000026 | Avilamycin\_A\_biosynthetic\_gene\_cluster | 33 | 39.9837464445 | 449 | 2.9e-125 |
| BAO66541.1 | type\_I\_polyketide\_synthase | BGC0000042 | Cremimycin\_biosynthetic\_gene\_cluster | 30 | 46.8508736286 | 448 | 3.8e-125 |
| AAW03325.1 | CtaB | BGC0000982 | Cystothiazole\_A\_biosynthetic\_gene\_cluster | 33 | 36.9362047948 | 448 | 3.8e-125 |
| ABJ97437.1 | MerA | BGC0001012 | Meridamycin\_biosynthetic\_gene\_cluster | 30 | 46.566436408 | 448 | 5e-125 |
| ADC79619.1 | BafAIV | BGC0000028 | Bafilomycin\_biosynthetic\_gene\_cluster | 29 | 45.9975619667 | 448 | 5e-125 |
| CAD29793.1 | polyketide\_synthase\_type\_I | BGC0001015 | Microcystin\_biosynthetic\_gene\_cluster | 30 | 46.2819991873 | 448 | 5e-125 |
| AAC68815.1 | FK506\_polyketide\_synthase | BGC0000353 | FK506\_biosynthetic\_gene\_cluster | 30 | 45.2255180821 | 448 | 6.5e-125 |
| AMK48247.1 | 6-methylsalicylic\_acid\_synthase | BGC0001351\_c2 | Cahuitamycins\_biosynthetic\_gene\_cluster | 33 | 40.5119869972 | 447 | 8.5e-125 |
| AAP42874.1 | NanA8 | BGC0000105 | Nanchangmycin\_biosynthetic\_gene\_cluster | 30 | 46.9321414059 | 447 | 8.5e-125 |
| ADF88280.1 | polyketide\_synthase | BGC0000981 | Cylindrospermopsin\_biosynthetic\_gene\_cluster | 30 | 46.6477041853 | 447 | 8.5e-125 |
| CAQ18828.1 | polyketide\_synthase | BGC0000954 | Ajudazol\_biosynthetic\_gene\_cluster | 31 | 46.8915075173 | 447 | 8.5e-125 |
| ABB86410.1 | GelC | BGC0000067 | Geldanamycin\_biosynthetic\_gene\_cluster | 30 | 46.566436408 | 447 | 8.5e-125 |
| BAB69194.1 | modular\_polyketide\_synthase | BGC0000117 | Oligomycin\_biosynthetic\_gene\_cluster | 30 | 46.2413652987 | 446 | 1.5e-124 |
| CAQ64686.1 | lasalocid\_modular\_polyketide\_synthase | BGC0000087 | Lasalocid\_biosynthetic\_gene\_cluster | 30 | 46.1600975213 | 446 | 1.9e-124 |
| BAQ21939.1 | putative\_type\_I\_polyketide\_synthase | BGC0001204 | Versipelostatin\_biosynthetic\_gene\_cluster | 30 | 47.2165786266 | 446 | 1.9e-124 |
| ABV83221.1 | CppI | BGC0000116 | Nystatin-like\_Pseudonocardia\_polyene\_biosynthetic\_gene\_cluster | 33 | 36.7330353515 | 446 | 1.9e-124 |
| CAD19086.1 | StiB\_protein | BGC0000153 | Stigmatellin\_biosynthetic\_gene\_cluster | 34 | 37.0174725721 | 446 | 2.5e-124 |
| AAM77986.1 | iterative\_type\_I\_polyketide\_synthase | BGC0000112 | Neocarzinostatin\_biosynthetic\_gene\_cluster | 31 | 42.1373425437 | 446 | 2.5e-124 |
| ABK32288.1 | JerB | BGC0000080 | Jerangolid\_biosynthetic\_gene\_cluster | 34 | 36.9362047948 | 446 | 2.5e-124 |
| ADZ24998.1 | polyketide\_synthase | BGC0000380 | Leupyrrin\_biosynthetic\_gene\_cluster | 30 | 50.9955302722 | 445 | 4.2e-124 |
| AAC46026.1 | polyketide\_synthase\_modules\_4\_and\_5 | BGC0000113 | Niddamycin\_biosynthetic\_gene\_cluster | 30 | 47.2165786266 | 445 | 4.2e-124 |
| BAG85026.1 | putative\_polyketide\_synthase | BGC0000086 | Lasalocid\_biosynthetic\_gene\_cluster | 30 | 46.1600975213 | 445 | 4.2e-124 |
| CUW01191.1 | Type\_I\_polyketide\_synthase | BGC0001359\_c2 | PM100117\_/\_PM100118\_biosynthetic\_gene\_cluster | 31 | 47.6635514019 | 445 | 4.2e-124 |
| CQR60497.1 | Polyketide\_synthase,\_type\_I,\_modules:\_loading,\_1,\_2\_and\_3 | BGC0001287 | Chaxamycin\_A\_/\_chaxamycin\_B\_/\_chaxamycin\_C\_/\_chaxamycin\_D | 33 | 39.3742381146 | 445 | 4.2e-124 |
| AFL48527.1 | laidlomycin\_polyketide\_synthase\_(module\_3\_and\_module\_4) | BGC0000084 | Laidlomycin\_biosynthetic\_gene\_cluster | 30 | 46.688338074 | 445 | 4.2e-124 |
| ABK32289.1 | JerC | BGC0000080 | Jerangolid\_biosynthetic\_gene\_cluster | 33 | 36.8549370175 | 445 | 4.2e-124 |
| AAC46025.1 | polyketide\_synthase\_module\_3 | BGC0000113 | Niddamycin\_biosynthetic\_gene\_cluster | 30 | 46.20073141 | 445 | 5.5e-124 |
| ACN69988.1 | polyketide\_synthase | BGC0000079 | Indanomycin\_biosynthetic\_gene\_cluster | 30 | 48.5168630638 | 444 | 7.2e-124 |
| CAD19087.1 | StiC\_protein | BGC0000153 | Stigmatellin\_biosynthetic\_gene\_cluster | 30 | 47.4603819586 | 444 | 7.2e-124 |
| AEH42491.1 | polyketide\_synthase | BGC0000032 | Calcimycin\_biosynthetic\_gene\_cluster | 29 | 45.956928078 | 444 | 7.2e-124 |
| ABB88521.1 | polyketide\_synthase\_type\_I | BGC0000050 | E-837\_biosynthetic\_gene\_cluster | 30 | 46.6477041853 | 444 | 9.4e-124 |
| ABC84460.1 | NigAV | BGC0000114 | Nigericin\_biosynthetic\_gene\_cluster | 29 | 46.322633076 | 444 | 9.4e-124 |
| ABX60162.1 | polyketide\_synthase | BGC0000978 | Cylindrospermopsin\_biosynthetic\_gene\_cluster | 30 | 46.6477041853 | 444 | 9.4e-124 |
| ABW96540.1 | type\_I\_modular\_polyketide\_synthase | BGC0000159 | Tautomycin\_biosynthetic\_gene\_cluster | 30 | 45.956928078 | 444 | 9.4e-124 |
| AEE88284.1 | CurF | BGC0000976 | Curacin\_biosynthetic\_gene\_cluster | 29 | 47.0946769606 | 444 | 9.4e-124 |
| AAT70101.1 | CurF | BGC0001165 | Curacin\_biosynthetic\_gene\_cluster | 29 | 47.0946769606 | 443 | 1.2e-123 |
| ABB05102.1 | LipPks1 | BGC0001003 | Lipomycin\_biosynthetic\_gene\_cluster | 34 | 36.9768386835 | 443 | 1.2e-123 |
| BAR73020.1 | putative\_PKS\_(KS-AT-DH-KR-ACP-KS-AT-DH-KR-ACP-KS-AT-DH-KR-ACP) | BGC0001194 | Hitachimycin\_biosynthetic\_gene\_cluster | 29 | 46.1194636327 | 443 | 1.2e-123 |
| ADC45534.1 | modular\_polyketide\_synthase | BGC0000093 | Meilingmycin\_biosynthetic\_gene\_cluster | 29 | 47.4603819586 | 443 | 1.2e-123 |
| AAC69330.1 | type\_I\_polyketide\_synthase\_PikAII | BGC0000094 | Methymycin\_/\_pikromycin\_biosynthetic\_gene\_cluster | 29 | 52.2551808208 | 443 | 1.6e-123 |
| ADU86003.1 | putative\_modular\_polyketide\_synthase | BGC0000165 | Tiacumicin\_B\_biosynthetic\_gene\_cluster | 31 | 45.3067858594 | 443 | 1.6e-123 |
| BAT51066.1 | type\_I\_polyketide\_synthase | BGC0001296 | Streptazone\_E\_biosynthetic\_gene\_cluster | 32 | 41.2840308818 | 443 | 2.1e-123 |
| AEZ64504.1 | Herc | BGC0001065 | Herboxidiene\_biosynthetic\_gene\_cluster | 30 | 49.1670052824 | 443 | 2.1e-123 |
| AAT70096.1 | CurA | BGC0001165 | Curacin\_biosynthetic\_gene\_cluster | 33 | 37.2206420154 | 442 | 2.7e-123 |
| CAM00062.1 | EryAI\_Erythromycin\_polyketide\_synthase\_modules\_1\_and\_2 | BGC0000055 | Erythromycin\_biosynthetic\_gene\_cluster | 34 | 36.9362047948 | 442 | 2.7e-123 |
| AEE88289.1 | CurA | BGC0000976 | Curacin\_biosynthetic\_gene\_cluster | 33 | 37.2206420154 | 442 | 2.7e-123 |
| ABV97154.1 | Beta-ketoacyl\_synthase | BGC0000137 | Rifamycin\_biosynthetic\_gene\_cluster | 29 | 45.956928078 | 442 | 3.6e-123 |
| BAE93731.1 | type\_I\_polyketide\_synthase | BGC0000164 | Tetronomycin\_biosynthetic\_gene\_cluster | 30 | 46.1600975213 | 442 | 3.6e-123 |
| ACN69990.1 | polyketide\_synthase | BGC0000079 | Indanomycin\_biosynthetic\_gene\_cluster | 30 | 46.3632669646 | 442 | 3.6e-123 |
| ACB37741.1 | putative\_type\_I\_polyketide\_synthase | BGC0000162 | Tetrocarcin\_A\_biosynthetic\_gene\_cluster | 30 | 46.8102397399 | 441 | 6.1e-123 |
| AEZ53945.1 | polyketide\_synthase | BGC0000144 | Salinomycin\_biosynthetic\_gene\_cluster | 29 | 45.7943925234 | 441 | 6.1e-123 |
| ABK32258.1 | AmbD | BGC0000014 | Ambruticin\_biosynthetic\_gene\_cluster | 30 | 50.1015847217 | 441 | 6.1e-123 |
| AEC13070.1 | fosD | BGC0000060 | Fostriecin\_biosynthetic\_gene\_cluster | 34 | 36.7330353515 | 441 | 6.1e-123 |
| AKD43753.1 | HerB | BGC0001349 | Heronamide\_biosynthetic\_gene\_cluster | 30 | 46.7696058513 | 441 | 6.1e-123 |
| AAQ82564.1 | FscC | BGC0000061 | FR-008\_biosynthetic\_gene\_cluster | 30 | 46.20073141 | 441 | 8e-123 |
| ABW96541.1 | type\_I\_modular\_polyketide\_synthase | BGC0000159 | Tautomycin\_biosynthetic\_gene\_cluster | 29 | 49.0857375051 | 441 | 8e-123 |
| ABP55210.1 | beta-ketoacyl\_synthase | BGC0000142 | Salinilactam\_biosynthetic\_gene\_cluster | 30 | 47.1759447379 | 441 | 8e-123 |
| ctg1\_orf522 |  | BGC0001199 | Akaeolide\_biosynthetic\_gene\_cluster | 30 | 45.7943925234 | 441 | 8e-123 |
| CAA60459.1 | polyketide\_synthase | BGC0001040 | Rapamycin\_biosynthetic\_gene\_cluster | 30 | 46.078829744 | 440 | 1e-122 |
| BAR73007.1 | putative\_PKS\_(ACP-KS-AT-DH-KR-ACP-KS-AT-DH-ER-KR-ACP) | BGC0001194 | Hitachimycin\_biosynthetic\_gene\_cluster | 30 | 46.566436408 | 440 | 1e-122 |
| CAO98879.1 | polyketide\_synthase\_AufD | BGC0000023 | Aurafuron\_biosynthetic\_gene\_cluster | 33 | 39.9024786672 | 440 | 1e-122 |
| AAG23262.1 | polyketide\_synthase\_extender\_modules\_8-10 | BGC0000148 | Spinosad\_biosynthetic\_gene\_cluster | 29 | 46.1600975213 | 440 | 1e-122 |
| ABI93779.1 | GdmPKS | BGC0000068 | Geldanamycin\_biosynthetic\_gene\_cluster | 30 | 46.4039008533 | 440 | 1.4e-122 |
| AHE80994.1 | PieA4 | BGC0001169 | Piericidin\_A1\_biosynthetic\_gene\_cluster | 30 | 45.7943925234 | 440 | 1.4e-122 |
| BAD08359.1 | polyketide\_synthase\_modules\_5-6 | BGC0000167 | Vicenistatin\_biosynthetic\_gene\_cluster | 30 | 46.566436408 | 440 | 1.4e-122 |
| AFU82616.1 | polyketide\_synthase | BGC0000998 | Guadinomine\_biosynthetic\_gene\_cluster | 31 | 46.6070702966 | 440 | 1.8e-122 |
| AHH99926.1 | PKS\_I | BGC0000002 | Aculeximycin\_biosynthetic\_gene\_cluster | 29 | 48.3543275091 | 439 | 2.3e-122 |
| ACB46471.1 | polyketide\_synthase | BGC0000082 | Kijanimicin\_biosynthetic\_gene\_cluster | 30 | 47.0540430719 | 439 | 2.3e-122 |
| CQR60493.1 | Polyketide\_synthase,\_type\_I,\_modules:\_9\_and\_10 | BGC0001287 | Chaxamycin\_A\_/\_chaxamycin\_B\_/\_chaxamycin\_C\_/\_chaxamycin\_D | 31 | 46.7289719626 | 439 | 3e-122 |
| AAO06918.1 | GdmAIII | BGC0000066 | Geldanamycin\_biosynthetic\_gene\_cluster | 30 | 46.8102397399 | 438 | 4e-122 |
| CAL58685.1 | polyketide\_synthase | BGC0000149 | Spirangien\_biosynthetic\_gene\_cluster | 34 | 37.0174725721 | 438 | 4e-122 |
| AAO62584.1 | polyketide\_synthase\_type\_1 | BGC0001016 | Microcystin\_biosynthetic\_gene\_cluster | 31 | 46.2413652987 | 438 | 4e-122 |
| AAW03329.1 | CtaF | BGC0000982 | Cystothiazole\_A\_biosynthetic\_gene\_cluster | 34 | 36.8955709061 | 438 | 4e-122 |
| BAQ25511.1 | type\_I\_polyketide\_synthase | BGC0001288 | Maklamicin\_biosynthetic\_gene\_cluster | 30 | 46.2819991873 | 438 | 4e-122 |
| ACO94483.1 | polyketide\_synthase\_type\_I | BGC0000097 | ML-449\_biosynthetic\_gene\_cluster | 29 | 46.6070702966 | 438 | 4e-122 |
| BAQ21940.1 | putative\_Type\_I\_polyketide\_synthase | BGC0001204 | Versipelostatin\_biosynthetic\_gene\_cluster | 29 | 49.8577813897 | 438 | 4e-122 |
| AAS98781.1 | JamJ | BGC0001001 | Jamaicamide\_biosynthetic\_gene\_cluster | 29 | 47.2572125152 | 438 | 5.2e-122 |
| CUW01171.1 | Type\_I\_polyketide\_synthase | BGC0001359 | PM100117\_/\_PM100118\_biosynthetic\_gene\_cluster | 34 | 38.236489232 | 438 | 5.2e-122 |
| AAF71766.1 | NysI | BGC0000115 | Nystatin\_biosynthetic\_gene\_cluster | 33 | 36.8955709061 | 438 | 5.2e-122 |
| BAC68129.1 | modular\_polyketide\_synthase | BGC0000059 | Filipin\_biosynthetic\_gene\_cluster | 30 | 48.3949613978 | 438 | 5.2e-122 |
| AGI99497.1 | type\_I\_polyketide\_synthase | BGC0001004 | Lobophorin\_biosynthetic\_gene\_cluster | 33 | 38.0333197887 | 438 | 6.7e-122 |
| CAE45669.1 | borrelidin\_polyketide\_synthase,\_type\_I | BGC0000031 | Borrelidin\_biosynthetic\_gene\_cluster | 30 | 47.3791141812 | 438 | 6.7e-122 |
| AAG13919.1 | megalomicin\_6-deoxyerythronolide\_B\_synthase\_3 | BGC0000092 | Megalomicin\_biosynthetic\_gene\_cluster | 32 | 39.2523364486 | 437 | 8.8e-122 |
| BAH02269.1 | polyketide\_synthase | BGC0000126 | Pladienolide\_biosynthetic\_gene\_cluster | 28 | 54.8151158066 | 437 | 1.2e-121 |
| ABK32256.1 | AmbB | BGC0000014 | Ambruticin\_biosynthetic\_gene\_cluster | 34 | 37.1393742381 | 437 | 1.2e-121 |
| CAE46843.1 | Type\_I\_modular\_polyketide\_synthase | BGC0000103 | Mycolactone\_biosynthetic\_gene\_cluster | 29 | 47.5010158472 | 437 | 1.2e-121 |
| AHA12078.1 | polyketide\_synthase\_type\_1 | BGC0001172 | Chlorizidine\_A\_biosynthetic\_gene\_cluster | 35 | 36.8955709061 | 436 | 2e-121 |
| AHH99921.1 | PKS\_I | BGC0000002 | Aculeximycin\_biosynthetic\_gene\_cluster | 33 | 36.4485981308 | 436 | 2e-121 |
| CAQ18834.1 | polyketide\_synthase | BGC0000954 | Ajudazol\_biosynthetic\_gene\_cluster | 35 | 36.6517675742 | 436 | 2.6e-121 |
| ADF88276.1 | polyketide\_synthase | BGC0000981 | Cylindrospermopsin\_biosynthetic\_gene\_cluster | 29 | 47.1353108492 | 436 | 2.6e-121 |
| BAQ25512.1 | type\_I\_polyketide\_synthase | BGC0001288 | Maklamicin\_biosynthetic\_gene\_cluster | 30 | 51.6456724909 | 436 | 2.6e-121 |
| AAF71768.1 | NysK | BGC0000115 | Nystatin\_biosynthetic\_gene\_cluster | 31 | 45.1036164161 | 435 | 3.3e-121 |
| AAK57186.1 | MxaB2 | BGC0001022 | Myxalamid\_biosynthetic\_gene\_cluster | 34 | 35.4327509143 | 435 | 3.3e-121 |
| BAJ16468.1 | polyketide\_synthase | BGC0000058 | FD-891\_biosynthetic\_gene\_cluster | 30 | 46.9321414059 | 435 | 4.4e-121 |
| CAD55506.1 | putative\_type\_I\_polyketide\_synthase | BGC0000038 | Coelimycin\_biosynthetic\_gene\_cluster | 30 | 46.3632669646 | 435 | 4.4e-121 |
| ABV97151.1 | AMP-dependent\_synthetase\_and\_ligase | BGC0000137 | Rifamycin\_biosynthetic\_gene\_cluster | 33 | 39.9431125559 | 435 | 4.4e-121 |
| CAE46851.1 | Type\_I\_modular\_polyketide\_synthase | BGC0000103 | Mycolactone\_biosynthetic\_gene\_cluster | 29 | 47.7448191792 | 435 | 5.7e-121 |
| AAC69332.1 | type\_I\_polyketide\_synthase\_PikAIV | BGC0000094 | Methymycin\_/\_pikromycin\_biosynthetic\_gene\_cluster | 33 | 37.6676147907 | 435 | 5.7e-121 |
| ABV83229.1 | CppB | BGC0000116 | Nystatin-like\_Pseudonocardia\_polyene\_biosynthetic\_gene\_cluster | 34 | 36.6924014628 | 435 | 5.7e-121 |
| AEZ54377.1 | PieA4 | BGC0000124 | Piericidin\_A1\_biosynthetic\_gene\_cluster | 29 | 52.3770824868 | 435 | 5.7e-121 |
| AGY62754.1 | EbeB | BGC0000051 | Ebelactone\_biosynthetic\_gene\_cluster | 29 | 46.2413652987 | 434 | 7.5e-121 |
| AAS98782.1 | JamK | BGC0001001 | Jamaicamide\_biosynthetic\_gene\_cluster | 31 | 39.4555058919 | 434 | 7.5e-121 |
| AAZ77698.1 | ChlA5 | BGC0000036 | Chlorothricin\_biosynthetic\_gene\_cluster | 30 | 48.3949613978 | 434 | 7.5e-121 |
| AAC46027.1 | polyketide\_synthase\_module\_6 | BGC0000113 | Niddamycin\_biosynthetic\_gene\_cluster | 35 | 36.7736692401 | 434 | 7.5e-121 |
| AGC09484.1 | LobS1 | BGC0001183 | Lobophorin\_biosynthetic\_gene\_cluster | 33 | 38.114587566 | 434 | 7.5e-121 |
| AAQ90173.1 | polyketide\_synthase\_type\_I | BGC0000128 | Pyoluteorin\_biosynthetic\_gene\_cluster | 32 | 37.7895164567 | 434 | 9.7e-121 |
| BAP34734.1 | type\_I\_polyketide\_synthase | BGC0000078 | Incednine\_biosynthetic\_gene\_cluster | 30 | 47.0134091833 | 433 | 1.3e-120 |
| WP\_043476516.1 | beta-ketoacyl\_synthase | BGC0001232 | Leucanicidin\_biosynthetic\_gene\_cluster | 30 | 46.6477041853 | 433 | 1.3e-120 |
| WP\_048832936.1 | polyketide\_synthase | BGC0001348 | JBIR-100\_biosynthetic\_gene\_cluster | 33 | 38.3990247867 | 433 | 1.3e-120 |
| CAD89777.1 | MelF\_protein | BGC0001010 | Melithiazol\_biosynthetic\_gene\_cluster | 34 | 36.8549370175 | 433 | 1.3e-120 |
| WP\_063764078.1 | polyketide\_synthase | BGC0001348 | JBIR-100\_biosynthetic\_gene\_cluster | 30 | 46.6477041853 | 433 | 1.3e-120 |
| ABI94380.1 | tautomycetin\_biosynthetic\_PKS | BGC0000157 | Tautomycetin\_biosynthetic\_gene\_cluster | 29 | 46.9321414059 | 433 | 1.3e-120 |
| ctg1\_orf7 |  | BGC0000053 | Elaiophylin\_biosynthetic\_gene\_cluster | 31 | 42.218610321 | 433 | 1.7e-120 |
| BAK64637.1 | polyketide\_synthase | BGC0000135 | Reveromycin\_biosynthetic\_gene\_cluster | 29 | 47.9886225112 | 433 | 1.7e-120 |
| AAF71775.1 | NysB | BGC0000115 | Nystatin\_biosynthetic\_gene\_cluster | 33 | 37.0987403495 | 433 | 1.7e-120 |
| ABY66019.1 | 6-methylsalicylic\_acid\_synthase | BGC0001008 | Maduropeptin\_biosynthetic\_gene\_cluster | 33 | 40.8370581065 | 433 | 1.7e-120 |
| CUW01175.1 | Type\_I\_polyketide\_synthase | BGC0001359 | PM100117\_/\_PM100118\_biosynthetic\_gene\_cluster | 29 | 47.9886225112 | 433 | 2.2e-120 |
| CAD89773.1 | MelB\_protein | BGC0001009 | Melithiazol\_biosynthetic\_gene\_cluster | 33 | 36.8955709061 | 433 | 2.2e-120 |
| ABK32263.1 | AmbH | BGC0000014 | Ambruticin\_biosynthetic\_gene\_cluster | 32 | 37.3019097928 | 432 | 2.8e-120 |
| ABI94379.1 | tautomycetin\_biosynthetic\_PKS | BGC0000157 | Tautomycetin\_biosynthetic\_gene\_cluster | 29 | 47.6229175132 | 432 | 2.8e-120 |
| AAF00959.1 | McyD | BGC0001017 | Microcystin\_biosynthetic\_gene\_cluster | 30 | 46.20073141 | 432 | 2.8e-120 |
| AFV30250.1 | polyketide\_synthase | BGC0000075 | Hygrocin\_biosynthetic\_gene\_cluster | 28 | 56.6842746851 | 432 | 3.7e-120 |
| AEP40939.1 | polyketide\_synthase\_type\_I | BGC0000021 | Apoptolidin\_biosynthetic\_gene\_cluster | 30 | 46.1194636327 | 432 | 3.7e-120 |
| ADC79638.1 | TamAII | BGC0001052 | Tirandamycin\_biosynthetic\_gene\_cluster | 33 | 36.6924014628 | 432 | 3.7e-120 |
| CAE46850.1 | Type\_I\_modular\_polyketide\_synthase | BGC0000103 | Mycolactone\_biosynthetic\_gene\_cluster | 29 | 47.4197480699 | 432 | 3.7e-120 |
| ABV91287.1 | type\_I\_modular\_polyketide\_synthase | BGC0000158 | Tautomycetin\_biosynthetic\_gene\_cluster | 29 | 46.9321414059 | 432 | 3.7e-120 |
| CAI94682.1 | putative\_polyketide\_synthase | BGC0000141 | Rubradirin\_biosynthetic\_gene\_cluster | 33 | 37.5863470134 | 432 | 3.7e-120 |
| AAG23266.1 | polyketide\_synthase\_extender\_modules\_3-4 | BGC0000148 | Spinosad\_biosynthetic\_gene\_cluster | 33 | 37.0174725721 | 431 | 4.8e-120 |
| AEK75502.1 | type\_1\_polyketide\_synthase | BGC0000001 | Abyssomicin\_biosynthetic\_gene\_cluster | 30 | 47.0540430719 | 431 | 4.8e-120 |
| EYT83439.1 | beta-ketoacyl\_synthase | BGC0001213 | Nataxazole\_biosynthetic\_gene\_cluster | 32 | 39.9024786672 | 431 | 4.8e-120 |
| ABX60152.1 | polyketide\_synthase | BGC0000978 | Cylindrospermopsin\_biosynthetic\_gene\_cluster | 29 | 47.1353108492 | 431 | 6.3e-120 |
| AAX98185.1 | polyketide\_synthase\_type\_I | BGC0000052 | ECO-02301\_biosynthetic\_gene\_cluster | 29 | 54.8557496952 | 431 | 6.3e-120 |
| ABC84461.1 | NigAVI | BGC0000114 | Nigericin\_biosynthetic\_gene\_cluster | 34 | 37.7488825681 | 431 | 8.3e-120 |
| AAZ77673.1 | ChlB1 | BGC0000036 | Chlorothricin\_biosynthetic\_gene\_cluster | 32 | 40.8370581065 | 431 | 8.3e-120 |
| AAX98189.1 | polyketide\_synthase\_type\_I | BGC0000052 | ECO-02301\_biosynthetic\_gene\_cluster | 30 | 48.3949613978 | 431 | 8.3e-120 |
| AAM54075.1 | polyketide\_synthase | BGC0000020 | Ansamitocin\_biosynthetic\_gene\_cluster | 30 | 44.0877691995 | 431 | 8.3e-120 |
| AAZ94391.1 | modular\_polyketide\_synthase | BGC0000040 | Concanamycin\_A\_biosynthetic\_gene\_cluster | 29 | 46.566436408 | 430 | 1.1e-119 |
| ABV91286.1 | type\_I\_modular\_polyketide\_synthase | BGC0000158 | Tautomycetin\_biosynthetic\_gene\_cluster | 29 | 47.6229175132 | 430 | 1.1e-119 |
| EAU29808.1 | hypothetical\_protein | BGC0001400 | Citreoviridin\_biosynthetic\_gene\_cluster | 31 | 45.8756603007 | 430 | 1.1e-119 |
| AKD43761.1 | HerD | BGC0001349 | Heronamide\_biosynthetic\_gene\_cluster | 31 | 45.7943925234 | 430 | 1.1e-119 |
| ctg1\_orf521 |  | BGC0001199 | Akaeolide\_biosynthetic\_gene\_cluster | 28 | 48.5574969525 | 430 | 1.4e-119 |
| BAP34733.1 | type\_I\_polyketide\_synthase | BGC0000078 | Incednine\_biosynthetic\_gene\_cluster | 29 | 54.6932141406 | 430 | 1.4e-119 |
| CAQ18833.1 | polyketide\_synthase | BGC0000954 | Ajudazol\_biosynthetic\_gene\_cluster | 34 | 36.9362047948 | 430 | 1.8e-119 |
| BAT51065.1 | type\_I\_polyketide\_synthase | BGC0001296 | Streptazone\_E\_biosynthetic\_gene\_cluster | 29 | 46.2413652987 | 430 | 1.8e-119 |
| ACY06287.1 | type\_I\_polyketide\_synthase | BGC0001042 | Sanglifehrin\_A\_biosynthetic\_gene\_cluster | 34 | 36.5298659082 | 430 | 1.8e-119 |
| CAJ46689.1 | polyketide\_synthase | BGC0000969 | Chondramide\_biosynthetic\_gene\_cluster | 34 | 36.2860625762 | 430 | 1.8e-119 |
| ACB37740.1 | putative\_type\_I\_polyketide\_synthase | BGC0000162 | Tetrocarcin\_A\_biosynthetic\_gene\_cluster | 28 | 53.7180008127 | 429 | 2.4e-119 |
| AEZ53950.1 | polyketide\_synthase | BGC0000144 | Salinomycin\_biosynthetic\_gene\_cluster | 29 | 47.2572125152 | 429 | 2.4e-119 |
| BAQ21946.1 | putative\_type\_I\_polyketide\_synthase | BGC0001204 | Versipelostatin\_biosynthetic\_gene\_cluster | 29 | 46.6477041853 | 429 | 2.4e-119 |
| AAS79461.1 | polyketide\_synthase\_subunit | BGC0000035 | Chalcomycin\_biosynthetic\_gene\_cluster | 31 | 44.5347419748 | 429 | 2.4e-119 |
| ctg1\_orf10 |  | BGC0000053 | Elaiophylin\_biosynthetic\_gene\_cluster | 29 | 46.4851686306 | 429 | 2.4e-119 |
| CAA60460.1 | polyketide\_synthase | BGC0001040 | Rapamycin\_biosynthetic\_gene\_cluster | 30 | 45.6724908574 | 429 | 2.4e-119 |
| AAS98777.1 | JamE | BGC0001001 | Jamaicamide\_biosynthetic\_gene\_cluster | 33 | 37.0174725721 | 429 | 3.1e-119 |
| AAS98200.1 | MSAS-type\_polyketide\_synthase | BGC0001273 | Asperlactone\_biosynthetic\_gene\_cluster | 31 | 39.0491670053 | 429 | 3.1e-119 |
| ALP32043.1 | CycC | BGC0001293 | Cyclizidine\_biosynthetic\_gene\_cluster | 30 | 44.2096708655 | 429 | 3.1e-119 |
| ALA09355.1 | type\_I\_modular\_PKS | BGC0001303 | Lobosamide\_biosynthetic\_gene\_cluster | 34 | 36.4079642422 | 429 | 3.1e-119 |
| AEU17898.1 | putative\_type\_I\_PKS | BGC0001072 | Simocyclinone\_biosynthetic\_gene\_cluster | 33 | 37.1393742381 | 428 | 4.1e-119 |
| AAF86392.1 | FkbC | BGC0000994 | FK520\_biosynthetic\_gene\_cluster | 29 | 45.4693214141 | 428 | 4.1e-119 |
| AFL48533.1 | laidlomycin\_polyketide\_synthase\_(module\_10) | BGC0000084 | Laidlomycin\_biosynthetic\_gene\_cluster | 33 | 36.7330353515 | 428 | 4.1e-119 |
| AAK57190.1 | MxaF | BGC0001022 | Myxalamid\_biosynthetic\_gene\_cluster | 33 | 35.7171881349 | 428 | 5.3e-119 |
| AAM54078.1 | polyketide\_synthase | BGC0000020 | Ansamitocin\_biosynthetic\_gene\_cluster | 30 | 46.20073141 | 428 | 5.3e-119 |
| ACB46488.1 | polyketide\_synthase | BGC0000082 | Kijanimicin\_biosynthetic\_gene\_cluster | 33 | 36.8143031288 | 428 | 5.3e-119 |
| AAQ82565.1 | FscB | BGC0000034 | Candicidin\_biosynthetic\_gene\_cluster | 29 | 53.3929297034 | 428 | 5.3e-119 |
| AGC24270.1 | PrlP | BGC0001038 | Pyralomicin\_biosynthetic\_gene\_cluster | 33 | 36.9768386835 | 428 | 5.3e-119 |
| ADU86004.1 | putative\_modular\_polyketide\_synthase | BGC0000165 | Tiacumicin\_B\_biosynthetic\_gene\_cluster | 30 | 45.4693214141 | 428 | 7e-119 |
| ABK32291.1 | JerE | BGC0000080 | Jerangolid\_biosynthetic\_gene\_cluster | 33 | 36.5298659082 | 428 | 7e-119 |
| CAJ88187.2 | putative\_type\_I\_modular\_polyketide\_synthase | BGC0000151 | Stambomycin\_biosynthetic\_gene\_cluster | 29 | 47.7854530679 | 428 | 7e-119 |
| AAO65799.1 | monensin\_polyketide\_synthase\_modules\_5\_and\_6 | BGC0000100 | Monensin\_biosynthetic\_gene\_cluster | 30 | 46.566436408 | 427 | 9.1e-119 |
| AAX98186.1 | polyketide\_synthase\_type\_I | BGC0000052 | ECO-02301\_biosynthetic\_gene\_cluster | 29 | 46.8915075173 | 427 | 1.2e-118 |
| ACO94456.1 | polyketide\_synthase\_type\_I | BGC0000029 | BE-14106\_biosynthetic\_gene\_cluster | 29 | 48.1511580658 | 427 | 1.2e-118 |
| CAQ18838.1 | polyketide\_synthase | BGC0000954 | Ajudazol\_biosynthetic\_gene\_cluster | 34 | 36.1641609102 | 427 | 1.2e-118 |
| CAQ52624.1 | type\_I\_polyketide\_synthase,\_modules\_7-8 | BGC0001066 | Kendomycin\_biosynthetic\_gene\_cluster | 31 | 44.5347419748 | 426 | 1.6e-118 |
| ADN13832.1 | Beta-ketoacyl\_synthase | BGC0001164 | 1-heptadecene\_biosynthetic\_gene\_cluster | 32 | 36.5298659082 | 426 | 1.6e-118 |
| AHH99920.1 | PKS\_I | BGC0000002 | Aculeximycin\_biosynthetic\_gene\_cluster | 32 | 35.7171881349 | 426 | 2e-118 |
| CAJ88186.1 | putative\_modular\_polyketide\_synthase | BGC0000151 | Stambomycin\_biosynthetic\_gene\_cluster | 30 | 46.1194636327 | 426 | 2e-118 |
| AGI61662.1 | modular\_PKS | BGC0000095\_c5 | Micromonolactam\_biosynthetic\_gene\_cluster | 34 | 36.4892320195 | 426 | 2e-118 |
| BAH02270.1 | polyketide\_synthase | BGC0000126 | Pladienolide\_biosynthetic\_gene\_cluster | 33 | 36.6517675742 | 426 | 2e-118 |
| BAD08373.1 | polyketide\_synthase\_modules\_1-3 | BGC0000167 | Vicenistatin\_biosynthetic\_gene\_cluster | 29 | 45.6724908574 | 426 | 2.7e-118 |
| BAQ25483.1 | type\_I\_polyketide\_synthase | BGC0001288 | Maklamicin\_biosynthetic\_gene\_cluster | 33 | 36.6924014628 | 426 | 2.7e-118 |
| ACF35446.1 | MbcAII | BGC0000090 | Macbecin\_biosynthetic\_gene\_cluster | 29 | 46.6070702966 | 426 | 2.7e-118 |
| CAO85897.1 | modular\_polyketide\_synthase\_NorB | BGC0000110 | Neoaureothin\_biosynthetic\_gene\_cluster | 29 | 47.0540430719 | 425 | 3.5e-118 |
| CAM00064.1 | EryAII\_Erythromycin\_polyketide\_synthase\_modules\_3\_and\_4 | BGC0000055 | Erythromycin\_biosynthetic\_gene\_cluster | 32 | 36.8955709061 | 425 | 3.5e-118 |
| ALP32046.1 | CycF | BGC0001293 | Cyclizidine\_biosynthetic\_gene\_cluster | 29 | 46.8102397399 | 425 | 4.5e-118 |
| AAF19814.1 | MtaF | BGC0001024 | Myxothiazol\_biosynthetic\_gene\_cluster | 33 | 40.3088175538 | 425 | 4.5e-118 |
| CCP20049.1 | divL2\_protein | BGC0001119 | Divergolide\_biosynthetic\_gene\_cluster | 28 | 55.1401869159 | 425 | 4.5e-118 |
| AAU93806.2 | polyketide\_synthase\_modules\_3\_and\_4 | BGC0000054 | Erythromycin\_biosynthetic\_gene\_cluster | 32 | 36.6517675742 | 425 | 4.5e-118 |
| AGY62755.1 | EbeC | BGC0000051 | Ebelactone\_biosynthetic\_gene\_cluster | 32 | 36.6517675742 | 425 | 5.9e-118 |
| AIT55262.1 | GulE | BGC0000072 | Gulmirecin\_biosynthetic\_gene\_cluster | 33 | 36.6924014628 | 425 | 5.9e-118 |
| CAN89636.1 | putative\_polyketide\_synthase | BGC0001070 | Kirromycin\_biosynthetic\_gene\_cluster | 29 | 46.3632669646 | 425 | 5.9e-118 |
| BAE93730.1 | type\_I\_polyketide\_synthase | BGC0000164 | Tetronomycin\_biosynthetic\_gene\_cluster | 30 | 47.2572125152 | 425 | 5.9e-118 |
| ADX66461.1 | ScnS2 | BGC0000108 | Natamycin\_biosynthetic\_gene\_cluster | 33 | 37.2206420154 | 425 | 5.9e-118 |
| ADF88275.1 | polyketide\_synthase | BGC0000981 | Cylindrospermopsin\_biosynthetic\_gene\_cluster | 32 | 36.6517675742 | 425 | 5.9e-118 |
| ABY21540.1 | AngAIII | BGC0000018 | Angolamycin\_biosynthetic\_gene\_cluster | 29 | 48.4762291751 | 424 | 7.7e-118 |
| AAZ77697.1 | ChlA4 | BGC0000036 | Chlorothricin\_biosynthetic\_gene\_cluster | 30 | 46.322633076 | 424 | 1e-117 |
| ABV83223.1 | CppK | BGC0000116 | Nystatin-like\_Pseudonocardia\_polyene\_biosynthetic\_gene\_cluster | 32 | 39.5774075579 | 424 | 1e-117 |
| ACO94484.1 | polyketide\_synthase\_type\_I | BGC0000097 | ML-449\_biosynthetic\_gene\_cluster | 31 | 42.5436814303 | 424 | 1e-117 |
| AAC69329.1 | type\_I\_polyketide\_synthase\_PikAI | BGC0000094 | Methymycin\_/\_pikromycin\_biosynthetic\_gene\_cluster | 33 | 37.3019097928 | 424 | 1e-117 |
| AAC01714.1 | RifE | BGC0000136 | Rifamycin\_biosynthetic\_gene\_cluster | 30 | 45.59122308 | 423 | 1.3e-117 |
| CCP20051.1 | divM\_protein | BGC0001119 | Divergolide\_biosynthetic\_gene\_cluster | 30 | 46.7696058513 | 423 | 1.3e-117 |
| AGI99482.1 | Type\_I\_polyketide\_synthase | BGC0001004 | Lobophorin\_biosynthetic\_gene\_cluster | 30 | 46.7289719626 | 423 | 1.3e-117 |
| CUW01172.1 | Type\_I\_polyketide\_synthase | BGC0001359 | PM100117\_/\_PM100118\_biosynthetic\_gene\_cluster | 33 | 36.2454286875 | 423 | 2.2e-117 |
| CCE88377.1 | non-ribosomal\_peptide\_synthetase/polyketide\_synthase | BGC0001034 | Pellasoren\_biosynthetic\_gene\_cluster | 33 | 35.9203575782 | 423 | 2.2e-117 |
| AFL48525.1 | laidlomycin\_polyketide\_synthase\_(loading\_module\_and\_module\_1) | BGC0000084 | Laidlomycin\_biosynthetic\_gene\_cluster | 29 | 49.2482730597 | 423 | 2.2e-117 |
| ABO15860.1 | polyketide\_synthase | BGC0000130 | Pyrrolomycin\_biosynthetic\_gene\_cluster | 33 | 36.8549370175 | 423 | 2.2e-117 |
| ABV83230.1 | CppC | BGC0000116 | Nystatin-like\_Pseudonocardia\_polyene\_biosynthetic\_gene\_cluster | 30 | 46.4039008533 | 423 | 2.2e-117 |
| AAO65798.1 | monensin\_polyketide\_synthase\_modules\_3\_and\_4 | BGC0000100 | Monensin\_biosynthetic\_gene\_cluster | 30 | 46.444534742 | 423 | 2.2e-117 |
| CAQ64689.1 | lasalocid\_modular\_polyketide\_synthase | BGC0000087 | Lasalocid\_biosynthetic\_gene\_cluster | 33 | 36.5298659082 | 422 | 2.9e-117 |
| AHH99925.1 | PKS\_I | BGC0000002 | Aculeximycin\_biosynthetic\_gene\_cluster | 32 | 36.4079642422 | 422 | 2.9e-117 |
| BAG85029.1 | putative\_polyketide\_synthase | BGC0000086 | Lasalocid\_biosynthetic\_gene\_cluster | 33 | 36.5298659082 | 422 | 3.8e-117 |
| ANC94964.1 | AlmHIII | BGC0001396 | Aldgamycin\_biosynthetic\_gene\_cluster | 31 | 41.0402275498 | 422 | 3.8e-117 |
| AHH99924.1 | PKS\_I | BGC0000002 | Aculeximycin\_biosynthetic\_gene\_cluster | 29 | 46.4851686306 | 422 | 3.8e-117 |
| ACF35445.1 | MbcAI | BGC0000090 | Macbecin\_biosynthetic\_gene\_cluster | 29 | 52.5396180414 | 421 | 5e-117 |
| ABC84459.1 | NigAIV | BGC0000114 | Nigericin\_biosynthetic\_gene\_cluster | 28 | 47.1759447379 | 421 | 6.5e-117 |
| AAK73501.1 | AmphI | BGC0000015 | Amphotericin\_biosynthetic\_gene\_cluster | 32 | 36.8549370175 | 421 | 6.5e-117 |
| AFL48529.1 | laidlomycin\_polyketide\_synthase\_(module\_5\_and\_module\_6) | BGC0000084 | Laidlomycin\_biosynthetic\_gene\_cluster | 29 | 45.835026412 | 421 | 6.5e-117 |
| BAO66528.1 | type\_I\_polyketide\_synthase | BGC0000042 | Cremimycin\_biosynthetic\_gene\_cluster | 32 | 43.0719219829 | 421 | 6.5e-117 |
| CBA11583.1 | polyketide\_synthase\_type\_I | BGC0001046 | Streptolydigin\_biosynthetic\_gene\_cluster | 32 | 36.8549370175 | 421 | 8.5e-117 |
| ADC79616.1 | BafAI | BGC0000028 | Bafilomycin\_biosynthetic\_gene\_cluster | 32 | 36.7330353515 | 421 | 8.5e-117 |
| CBZ41585.1 | putative\_modular\_polyketide\_synthase | BGC0000151 | Stambomycin\_biosynthetic\_gene\_cluster | 30 | 46.1600975213 | 420 | 1.1e-116 |
| CAC20921.1 | PimS2\_protein | BGC0000125 | Pimaricin\_biosynthetic\_gene\_cluster | 33 | 37.0987403495 | 420 | 1.5e-116 |
| ctg1\_orf523 |  | BGC0001199 | Akaeolide\_biosynthetic\_gene\_cluster | 29 | 46.20073141 | 420 | 1.5e-116 |
| CAC22145.1 | putative\_type\_I\_polyketide\_synthase | BGC0000038 | Coelimycin\_biosynthetic\_gene\_cluster | 31 | 39.7805770012 | 420 | 1.5e-116 |
| AKD43768.1 | HerA1 | BGC0001349 | Heronamide\_biosynthetic\_gene\_cluster | 28 | 45.9975619667 | 420 | 1.9e-116 |
| AAD03047.1 | type\_I\_polyketide\_synthase | BGC0000041 | Coronafacic\_acid\_biosynthetic\_gene\_cluster | 30 | 46.7289719626 | 419 | 2.5e-116 |
| CUW01176.1 | Type\_I\_polyketide\_synthase | BGC0001359\_c2 | PM100117\_/\_PM100118\_biosynthetic\_gene\_cluster | 33 | 39.2929703373 | 419 | 3.2e-116 |
| AAC46024.1 | polyketide\_synthase\_modules\_1\_and\_2 | BGC0000113 | Niddamycin\_biosynthetic\_gene\_cluster | 33 | 36.4079642422 | 419 | 3.2e-116 |
| AAM54077.1 | polyketide\_synthase | BGC0000020 | Ansamitocin\_biosynthetic\_gene\_cluster | 33 | 36.3673303535 | 419 | 3.2e-116 |
| AAO62582.1 | polyketide\_synthase\_peptide\_sythetase\_fusion\_protein | BGC0001016 | Microcystin\_biosynthetic\_gene\_cluster | 33 | 37.6676147907 | 419 | 3.2e-116 |
| AAK73514.1 | AmphC | BGC0000015 | Amphotericin\_biosynthetic\_gene\_cluster | 29 | 46.7289719626 | 418 | 4.2e-116 |
| ctg1\_orf28 |  | BGC0000096 | Midecamycin\_biosynthetic\_gene\_cluster | 29 | 48.0698902885 | 418 | 4.2e-116 |
| AAY28227.1 | HbmAIII | BGC0000074 | Herbimycin\_biosynthetic\_gene\_cluster | 29 | 47.4197480699 | 418 | 5.5e-116 |
| AAM70355.1 | CalO5 | BGC0000033 | Calicheamicin\_biosynthetic\_gene\_cluster | 32 | 39.2117025599 | 418 | 5.5e-116 |
| AEZ54379.1 | PieA6 | BGC0000124 | Piericidin\_A1\_biosynthetic\_gene\_cluster | 33 | 36.3673303535 | 418 | 5.5e-116 |
| AAU04878.1 | polyketide\_synthase | BGC0000365 | Glycopeptidolipid\_biosynthetic\_gene\_cluster | 31 | 39.6993092239 | 418 | 7.2e-116 |
| ADZ24996.1 | polyketide\_synthase | BGC0000380 | Leupyrrin\_biosynthetic\_gene\_cluster | 30 | 46.7696058513 | 418 | 7.2e-116 |
| AAQ84145.1 | Plm5 | BGC0000123 | Phoslactomycin\_B\_biosynthetic\_gene\_cluster | 30 | 47.0540430719 | 418 | 7.2e-116 |
| ADH04659.1 | TugC | BGC0001342 | Thuggacin\_biosynthetic\_gene\_cluster | 29 | 48.0292563998 | 418 | 7.2e-116 |
| ACN69992.1 | polyketide\_synthase | BGC0000079 | Indanomycin\_biosynthetic\_gene\_cluster | 32 | 37.2612759041 | 417 | 9.4e-116 |
| BAR73017.1 | putative\_PKS\_(KS-AT-KR-ACP-KS-AT-DH-KR-ACP) | BGC0001194 | Hitachimycin\_biosynthetic\_gene\_cluster | 30 | 45.8756603007 | 417 | 9.4e-116 |
| CAA60462.1 | polyketide\_synthase | BGC0001040 | Rapamycin\_biosynthetic\_gene\_cluster | 30 | 45.835026412 | 417 | 9.4e-116 |
| AGC09499.1 | LobS4 | BGC0001183 | Lobophorin\_biosynthetic\_gene\_cluster | 30 | 46.4851686306 | 417 | 9.4e-116 |
| AEZ54375.1 | PieA2 | BGC0000124 | Piericidin\_A1\_biosynthetic\_gene\_cluster | 33 | 36.3266964649 | 417 | 1.2e-115 |
| CAL58686.1 | polyketide\_synthase | BGC0000149 | Spirangien\_biosynthetic\_gene\_cluster | 30 | 44.2096708655 | 417 | 1.2e-115 |
| BAC57028.1 | protomycinolide\_IV\_synthase\_1 | BGC0000102 | Mycinamicin\_biosynthetic\_gene\_cluster | 32 | 36.2454286875 | 417 | 1.2e-115 |
| CAJ88177.1 | putative\_type\_I\_polyketide\_synthase | BGC0000151 | Stambomycin\_biosynthetic\_gene\_cluster | 31 | 44.9410808614 | 416 | 1.6e-115 |
| AAG23263.1 | polyketide\_synthase\_extender\_modules\_5-7 | BGC0000148 | Spinosad\_biosynthetic\_gene\_cluster | 29 | 46.7289719626 | 416 | 1.6e-115 |
| AEC13080.1 | fosB | BGC0000060 | Fostriecin\_biosynthetic\_gene\_cluster | 29 | 47.2572125152 | 416 | 1.6e-115 |
| AFL48532.1 | laidlomycin\_polyketide\_synthase\_(module\_11\_and\_module\_12) | BGC0000084 | Laidlomycin\_biosynthetic\_gene\_cluster | 30 | 47.4197480699 | 416 | 2.1e-115 |
| BAC68127.1 | modular\_polyketide\_synthase | BGC0000059 | Filipin\_biosynthetic\_gene\_cluster | 30 | 43.0312880943 | 416 | 2.1e-115 |
| ABC84456.1 | NigAI | BGC0000114 | Nigericin\_biosynthetic\_gene\_cluster | 34 | 36.8143031288 | 416 | 2.1e-115 |
| ctg1\_orf255 |  | BGC0001200 | Lorneic\_acid\_A\_biosynthetic\_gene\_cluster | 31 | 40.7964242178 | 416 | 2.1e-115 |
| AAF71776.1 | NysC | BGC0000115 | Nystatin\_biosynthetic\_gene\_cluster | 29 | 46.4851686306 | 416 | 2.1e-115 |
| AEZ54378.1 | PieA5 | BGC0000124 | Piericidin\_A1\_biosynthetic\_gene\_cluster | 30 | 46.9727752946 | 416 | 2.1e-115 |
| AAZ94388.1 | nodular\_polyketide\_synthase | BGC0000040 | Concanamycin\_A\_biosynthetic\_gene\_cluster | 33 | 36.7736692401 | 416 | 2.7e-115 |
| ACF35447.1 | MbcAIII | BGC0000090 | Macbecin\_biosynthetic\_gene\_cluster | 29 | 45.8756603007 | 416 | 2.7e-115 |
| CAQ52623.1 | type\_I\_polyketide\_synthase,\_module\_6 | BGC0001066 | Kendomycin\_biosynthetic\_gene\_cluster | 29 | 49.8984152783 | 415 | 3.6e-115 |
| AFL48528.1 | laidlomycin\_polyketide\_synthase\_(module\_7\_and\_module\_8) | BGC0000084 | Laidlomycin\_biosynthetic\_gene\_cluster | 29 | 45.5505891914 | 415 | 4.7e-115 |
| AAM81586.2 | putative\_type\_I\_polyketide\_synthase | BGC0000047 | Dihydrochalcomycin\_biosynthetic\_gene\_cluster | 28 | 54.164973588 | 415 | 4.7e-115 |
| ABX60153.1 | polyketide\_synthase | BGC0000978 | Cylindrospermopsin\_biosynthetic\_gene\_cluster | 32 | 36.3673303535 | 415 | 6.1e-115 |
| AEK75503.1 | type\_1\_polyketide\_synthase | BGC0000001 | Abyssomicin\_biosynthetic\_gene\_cluster | 32 | 36.4485981308 | 415 | 6.1e-115 |
| BAG85028.1 | putative\_polyketide\_synthase | BGC0000086 | Lasalocid\_biosynthetic\_gene\_cluster | 32 | 36.7736692401 | 415 | 6.1e-115 |
| CAQ64688.1 | lasalocid\_modular\_polyketide\_synthase | BGC0000087 | Lasalocid\_biosynthetic\_gene\_cluster | 32 | 36.7736692401 | 415 | 6.1e-115 |
| AKD43763.1 | HerG | BGC0001349 | Heronamide\_biosynthetic\_gene\_cluster | 30 | 46.3632669646 | 414 | 8e-115 |
| AHB82051.1 | polyketide\_synthase | BGC0001019 | Microsclerodermins\_biosynthetic\_gene\_cluster | 29 | 46.8508736286 | 414 | 8e-115 |
| ADC79639.1 | TamAIII | BGC0001052 | Tirandamycin\_biosynthetic\_gene\_cluster | 29 | 46.8915075173 | 414 | 8e-115 |
| AKD43769.1 | HerA2 | BGC0001349 | Heronamide\_biosynthetic\_gene\_cluster | 31 | 42.8687525396 | 414 | 1e-114 |
| ALP32045.1 | CycE | BGC0001293 | Cyclizidine\_biosynthetic\_gene\_cluster | 31 | 43.5595286469 | 414 | 1e-114 |
| BAF02925.1 | type\_I\_polyketide\_synthase | BGC0000073 | Halstoctacosanolide\_biosynthetic\_gene\_cluster | 33 | 37.5457131247 | 414 | 1e-114 |
| CAQ34918.1 | nonribosomal\_peptide\_synthetase/\_polyketide\_synthase | BGC0000986 | Dkxanthene\_biosynthetic\_gene\_cluster | 29 | 47.4603819586 | 414 | 1e-114 |
| AJO72736.1 | Type\_I\_modular\_polyketide\_synthase | BGC0001381 | Brasilinolide\_biosynthetic\_gene\_cluster | 32 | 36.5704997968 | 413 | 1.4e-114 |
| CAQ64692.1 | lasalocid\_modular\_polyketide\_synthase | BGC0000087 | Lasalocid\_biosynthetic\_gene\_cluster | 33 | 36.8549370175 | 413 | 1.8e-114 |
| ACZ65476.1 | type\_I\_modular\_polyketide\_synthase | BGC0000140 | RK-682\_biosynthetic\_gene\_cluster | 33 | 33.8480292564 | 413 | 1.8e-114 |
| AHH99922.1 | PKS\_I | BGC0000002 | Aculeximycin\_biosynthetic\_gene\_cluster | 33 | 36.0422592442 | 413 | 1.8e-114 |
| ABC84458.1 | NigAIII | BGC0000114 | Nigericin\_biosynthetic\_gene\_cluster | 29 | 46.8915075173 | 413 | 1.8e-114 |
| AAY28225.1 | HbmAI | BGC0000074 | Herbimycin\_biosynthetic\_gene\_cluster | 29 | 46.322633076 | 413 | 2.3e-114 |
| ABP55221.1 | acyl\_transferase\_domain\_protein | BGC0000142 | Salinilactam\_biosynthetic\_gene\_cluster | 32 | 36.8549370175 | 413 | 2.3e-114 |
| ANC94966.1 | AlmHI | BGC0001396 | Aldgamycin\_biosynthetic\_gene\_cluster | 32 | 36.9362047948 | 412 | 3e-114 |
| AHB82064.1 | polyketide\_synthase | BGC0001231 | Microsclerodermins\_biosynthetic\_gene\_cluster | 33 | 36.6111336855 | 412 | 3e-114 |
| AAP42857.1 | NanA3 | BGC0000105 | Nanchangmycin\_biosynthetic\_gene\_cluster | 29 | 48.0292563998 | 412 | 3e-114 |
| AAK73503.1 | AmphK | BGC0000015 | Amphotericin\_biosynthetic\_gene\_cluster | 31 | 39.5774075579 | 412 | 3e-114 |
| AFV30251.1 | polyketide\_synthase | BGC0000075 | Hygrocin\_biosynthetic\_gene\_cluster | 29 | 46.688338074 | 412 | 3e-114 |
| CAE45670.1 | borrelidin\_polyketide\_synthase,\_type\_I | BGC0000031 | Borrelidin\_biosynthetic\_gene\_cluster | 33 | 36.5704997968 | 412 | 4e-114 |
| ACB37743.1 | putative\_type\_I\_polyketide\_synthase | BGC0000162 | Tetrocarcin\_A\_biosynthetic\_gene\_cluster | 32 | 37.3019097928 | 411 | 5.2e-114 |
| AAG13918.1 | megalomicin\_6-deoxyerythronolide\_B\_synthase\_2 | BGC0000092 | Megalomicin\_biosynthetic\_gene\_cluster | 31 | 36.5704997968 | 411 | 5.2e-114 |
| BAB69192.1 | modular\_polyketide\_synthase | BGC0000117 | Oligomycin\_biosynthetic\_gene\_cluster | 33 | 36.2860625762 | 411 | 5.2e-114 |
| ACR33079.1 | polyketide\_synthase | BGC0000017 | Anatoxin\_biosynthetic\_gene\_cluster | 33 | 36.4485981308 | 411 | 6.8e-114 |
| AFV30249.1 | polyketide\_synthase | BGC0000075 | Hygrocin\_biosynthetic\_gene\_cluster | 29 | 46.322633076 | 411 | 6.8e-114 |
| ABO15861.1 | polyketide\_synthase | BGC0000130 | Pyrrolomycin\_biosynthetic\_gene\_cluster | 33 | 36.8143031288 | 411 | 8.8e-114 |
| ACY13415.1 | KR\_domain\_protein | BGC0001367 | Haliamide\_biosynthetic\_gene\_cluster | 33 | 36.4079642422 | 410 | 1.2e-113 |
| BAO66539.1 | type\_I\_polyketide\_synthase | BGC0000042 | Cremimycin\_biosynthetic\_gene\_cluster | 33 | 36.5298659082 | 410 | 1.5e-113 |
| ACV42478.1 | polyketide\_synthase | BGC0000043 | Curacin\_A\_biosynthetic\_gene\_cluster | 31 | 37.3831775701 | 410 | 1.5e-113 |
| AEE88277.1 | CurM | BGC0000976 | Curacin\_biosynthetic\_gene\_cluster | 31 | 37.3831775701 | 410 | 1.5e-113 |
| AEZ53952.1 | polyketide\_synthase | BGC0000144 | Salinomycin\_biosynthetic\_gene\_cluster | 28 | 46.078829744 | 410 | 1.5e-113 |
| AAT70108.1 | CurM | BGC0001165 | Curacin\_biosynthetic\_gene\_cluster | 31 | 37.3831775701 | 410 | 1.5e-113 |
| CAD29794.1 | peptide\_synthetase | BGC0001015 | Microcystin\_biosynthetic\_gene\_cluster | 32 | 36.8549370175 | 410 | 2e-113 |
| AEH42490.1 | polyketide\_synthase | BGC0000032 | Calcimycin\_biosynthetic\_gene\_cluster | 33 | 36.8143031288 | 410 | 2e-113 |
| AAB08104.3 | polyketide\_synthase | BGC0000155\_c3 | T-toxin\_biosynthetic\_gene\_cluster | 26 | 56.2779357985 | 409 | 2.6e-113 |
| CUI25744.1 | Polyketide\_synthase/Nonribosomal\_peptide\_synthase | BGC0001354 | U-68204\_biosynthetic\_gene\_cluster | 29 | 46.444534742 | 409 | 2.6e-113 |
| CUI25672.1 | Thiolactomycin\_Hybrid\_PKS-NRPS\_system | BGC0001353 | Thiolactomycin\_biosynthetic\_gene\_cluster | 30 | 45.7943925234 | 409 | 2.6e-113 |
| BAD38873.1 | polyketide\_synthase | BGC0000111 | Neocarzilin\_biosynthetic\_gene\_cluster | 30 | 48.0292563998 | 409 | 2.6e-113 |
| AMB48441.1 | polyketide\_synthase | BGC0001357 | Carbamidocyclophane\_biosynthetic\_gene\_cluster | 32 | 38.480292564 | 409 | 3.4e-113 |
| ctg1\_orf27 |  | BGC0000096 | Midecamycin\_biosynthetic\_gene\_cluster | 29 | 47.5416497359 | 409 | 3.4e-113 |
| ABP55222.1 | beta-ketoacyl\_synthase | BGC0000142 | Salinilactam\_biosynthetic\_gene\_cluster | 33 | 36.8549370175 | 409 | 3.4e-113 |
| AAY89049.1 | polyketide\_synthase | BGC0001069 | Chivosazole\_biosynthetic\_gene\_cluster | 34 | 35.5140186916 | 409 | 3.4e-113 |
| ACB46192.1 | polyketide\_synthase | BGC0000989 | Epothilone\_biosynthetic\_gene\_cluster | 33 | 36.9768386835 | 408 | 4.4e-113 |
| AJO72735.1 | Type\_I\_modular\_polyketide\_synthase | BGC0001381 | Brasilinolide\_biosynthetic\_gene\_cluster | 33 | 36.8955709061 | 408 | 4.4e-113 |
| ACO94470.1 | polyketide\_synthase\_type\_I | BGC0000029 | BE-14106\_biosynthetic\_gene\_cluster | 30 | 45.956928078 | 408 | 4.4e-113 |
| AAF62880.1 | EpoA | BGC0000991 | Epothilone\_biosynthetic\_gene\_cluster | 33 | 36.9768386835 | 408 | 4.4e-113 |
| AJO72737.1 | Type\_I\_modular\_polyketide\_synthase | BGC0001381 | Brasilinolide\_biosynthetic\_gene\_cluster | 29 | 46.7696058513 | 408 | 5.7e-113 |
| ALJ49921.1 | TtmH | BGC0001236 | Thiotetroamide\_biosynthetic\_gene\_cluster | 29 | 46.6477041853 | 408 | 5.7e-113 |
| BAC57032.1 | protomycinolide\_IV\_synthase\_5 | BGC0000102 | Mycinamicin\_biosynthetic\_gene\_cluster | 29 | 47.0540430719 | 408 | 5.7e-113 |
| AFV96142.1 | polyketide\_synthase | BGC0001064 | Cylindrocyclophane\_biosynthetic\_gene\_cluster | 33 | 37.0581064608 | 408 | 5.7e-113 |
| CAL58683.1 | polyketide\_synthase | BGC0000149 | Spirangien\_biosynthetic\_gene\_cluster | 32 | 35.6765542462 | 408 | 7.5e-113 |
| CAA16183.1 | polyketide\_synthase | BGC0001063 | Undecylprodigiosin\_biosynthetic\_gene\_cluster | 32 | 35.6765542462 | 407 | 9.8e-113 |
| AFP87523.1 | type\_I\_polyketide\_synthase | BGC0001159 | Marinopyrrole\_biosynthetic\_gene\_cluster | 33 | 37.0174725721 | 407 | 9.8e-113 |
| ctg1\_orf9 |  | BGC0000053 | Elaiophylin\_biosynthetic\_gene\_cluster | 32 | 36.8143031288 | 407 | 9.8e-113 |
| AAZ77699.1 | ChlA6 | BGC0000036 | Chlorothricin\_biosynthetic\_gene\_cluster | 33 | 36.8549370175 | 407 | 1.3e-112 |
| ctg1\_orf30 |  | BGC0000096 | Midecamycin\_biosynthetic\_gene\_cluster | 32 | 36.8955709061 | 406 | 1.7e-112 |
| BAF85837.1 | modular\_polyketide\_synthase | BGC0000109 | Nemadectin\_biosynthetic\_gene\_cluster | 29 | 47.5010158472 | 406 | 1.7e-112 |
| AEC13079.1 | fosA | BGC0000060 | Fostriecin\_biosynthetic\_gene\_cluster | 29 | 47.3384802926 | 406 | 2.2e-112 |
| BAK64650.1 | polyketide\_synthase | BGC0000135 | Reveromycin\_biosynthetic\_gene\_cluster | 33 | 36.1235270215 | 406 | 2.2e-112 |
| CAE02605.1 | polyketide\_synthase\_type\_I | BGC0000024 | Aureothin\_biosynthetic\_gene\_cluster | 29 | 46.4851686306 | 406 | 2.8e-112 |
| ADB12488.1 | EpoA | BGC0000990 | Epothilone\_biosynthetic\_gene\_cluster | 33 | 36.9768386835 | 406 | 2.8e-112 |
| ACO94498.1 | polyketide\_synthase\_type\_I | BGC0000097 | ML-449\_biosynthetic\_gene\_cluster | 30 | 46.2819991873 | 406 | 2.8e-112 |
| BAJ16470.1 | polyketide\_synthase | BGC0000058 | FD-891\_biosynthetic\_gene\_cluster | 30 | 48.2324258432 | 405 | 3.7e-112 |
| AAQ90174.1 | polyketide\_synthase\_type\_I | BGC0000128 | Pyoluteorin\_biosynthetic\_gene\_cluster | 32 | 37.8707842341 | 405 | 4.8e-112 |
| BAC68125.1 | modular\_polyketide\_synthase | BGC0000059 | Filipin\_biosynthetic\_gene\_cluster | 33 | 37.0174725721 | 405 | 4.8e-112 |
| ALA09356.1 | type\_I\_modular\_PKS | BGC0001303 | Lobosamide\_biosynthetic\_gene\_cluster | 33 | 36.8143031288 | 405 | 4.8e-112 |
| AAU93805.2 | polyketide\_synthase\_modules\_5\_and\_6 | BGC0000054 | Erythromycin\_biosynthetic\_gene\_cluster | 30 | 37.9114181227 | 404 | 6.3e-112 |
| ABX60161.1 | mixed\_NRPS/PKS | BGC0000978 | Cylindrospermopsin\_biosynthetic\_gene\_cluster | 29 | 45.8756603007 | 404 | 1.1e-111 |
| AEU17897.1 | putative\_type\_I\_PKS | BGC0001072 | Simocyclinone\_biosynthetic\_gene\_cluster | 30 | 47.5010158472 | 404 | 1.1e-111 |
| AAO65807.1 | monensin\_polyketide\_synthase\_module\_10 | BGC0000100 | Monensin\_biosynthetic\_gene\_cluster | 32 | 36.8955709061 | 403 | 1.4e-111 |
| BAC68126.1 | modular\_polyketide\_synthase | BGC0000059 | Filipin\_biosynthetic\_gene\_cluster | 32 | 36.9362047948 | 403 | 1.4e-111 |
| ADF88279.1 | mixed\_NRPS/PKS | BGC0000981 | Cylindrospermopsin\_biosynthetic\_gene\_cluster | 29 | 45.8756603007 | 403 | 1.8e-111 |
| AFU82617.1 | polyketide\_synthase | BGC0000998 | Guadinomine\_biosynthetic\_gene\_cluster | 33 | 36.9362047948 | 403 | 1.8e-111 |
| AAU93807.2 | polyketide\_synthase\_modules\_1\_and\_2 | BGC0000054 | Erythromycin\_biosynthetic\_gene\_cluster | 32 | 36.7330353515 | 403 | 1.8e-111 |
| BAC76493.1 | lankamycin\_synthase\_LkmAI | BGC0000085 | Lankamycin\_biosynthetic\_gene\_cluster | 32 | 37.5457131247 | 403 | 1.8e-111 |
| BAO66519.1 | type\_I\_polyketide\_synthase | BGC0000042 | Cremimycin\_biosynthetic\_gene\_cluster | 29 | 48.2324258432 | 403 | 2.4e-111 |
| AAQ82561.1 | FscA | BGC0000034 | Candicidin\_biosynthetic\_gene\_cluster | 33 | 36.8549370175 | 402 | 3.1e-111 |
| AHA38199.1 | GphF | BGC0000069 | Gephyronic\_acid\_biosynthetic\_gene\_cluster | 28 | 46.3632669646 | 402 | 3.1e-111 |
| CAE02606.1 | polyketide\_synthase\_type\_I | BGC0000024 | Aureothin\_biosynthetic\_gene\_cluster | 31 | 37.1393742381 | 402 | 4.1e-111 |
| AAB66507.1 | tylactone\_synthase\_module\_6 | BGC0000166 | Tylactone\_biosynthetic\_gene\_cluster | 32 | 37.2612759041 | 401 | 5.4e-111 |
| AAC69331.1 | type\_I\_polyketide\_synthase\_PikAIII | BGC0000094 | Methymycin\_/\_pikromycin\_biosynthetic\_gene\_cluster | 31 | 37.0174725721 | 401 | 5.4e-111 |
| CAD19093.1 | StiJ\_protein | BGC0000153 | Stigmatellin\_biosynthetic\_gene\_cluster | 32 | 36.8955709061 | 401 | 5.4e-111 |
| AFU82614.1 | mixed\_NRPS\_PKS | BGC0000998 | Guadinomine\_biosynthetic\_gene\_cluster | 30 | 46.444534742 | 401 | 5.4e-111 |
| AAZ94387.1 | modular\_polyketide\_synthase | BGC0000040 | Concanamycin\_A\_biosynthetic\_gene\_cluster | 33 | 35.4733848029 | 401 | 7e-111 |
| AAF26919.1 | polyketide\_synthase | BGC0000988 | Epothilone\_biosynthetic\_gene\_cluster | 32 | 37.0174725721 | 401 | 9.1e-111 |
| AHE80996.1 | PieA6 | BGC0001169 | Piericidin\_A1\_biosynthetic\_gene\_cluster | 33 | 37.1800081268 | 401 | 9.1e-111 |
| AAO65806.1 | monensin\_polyketide\_synthase\_modules\_11\_and\_12 | BGC0000100 | Monensin\_biosynthetic\_gene\_cluster | 28 | 52.6208858188 | 400 | 1.2e-110 |
| BAG85032.1 | putative\_polyketide\_synthase | BGC0000086 | Lasalocid\_biosynthetic\_gene\_cluster | 32 | 36.6111336855 | 400 | 1.2e-110 |
| AGC24271.1 | PrlQ | BGC0001038 | Pyralomicin\_biosynthetic\_gene\_cluster | 32 | 36.4892320195 | 400 | 1.2e-110 |
| ABV83228.1 | CppA | BGC0000116 | Nystatin-like\_Pseudonocardia\_polyene\_biosynthetic\_gene\_cluster | 32 | 35.7171881349 | 400 | 1.2e-110 |
| AAO65800.1 | monensin\_polyketide\_synthase\_modules\_7\_and\_8 | BGC0000100 | Monensin\_biosynthetic\_gene\_cluster | 29 | 46.4039008533 | 400 | 1.2e-110 |
| ABV99085.1 | thioester\_reductase\_domain | BGC0001007 | Lymphostin\_biosynthetic\_gene\_cluster | 33 | 36.6924014628 | 400 | 1.6e-110 |
| AEZ53953.1 | polyketide\_synthase | BGC0000144 | Salinomycin\_biosynthetic\_gene\_cluster | 31 | 37.0987403495 | 400 | 1.6e-110 |
| ctg1\_orf23 |  | BGC0001013 | Meridamycin\_biosynthetic\_gene\_cluster | 29 | 46.4039008533 | 399 | 2e-110 |
| ABC84469.1 | NigAIX | BGC0000114 | Nigericin\_biosynthetic\_gene\_cluster | 28 | 46.9321414059 | 399 | 2e-110 |
| ABC87512.1 | polyketide\_synthase | BGC0001011 | Meridamycin\_biosynthetic\_gene\_cluster | 29 | 46.4039008533 | 399 | 2e-110 |
| AAC46028.1 | polyketide\_synthase\_module\_7 | BGC0000113 | Niddamycin\_biosynthetic\_gene\_cluster | 33 | 36.7736692401 | 399 | 2.7e-110 |
| AAQ82568.1 | FscD | BGC0000061 | FR-008\_biosynthetic\_gene\_cluster | 33 | 37.1393742381 | 398 | 4.5e-110 |
| AEZ53951.1 | polyketide\_synthase | BGC0000144 | Salinomycin\_biosynthetic\_gene\_cluster | 32 | 36.3673303535 | 398 | 4.5e-110 |
| ADB23403.1 | polyketide\_synthase\_type\_I | BGC0001062 | TLN-05220\_biosynthetic\_gene\_cluster | 28 | 46.7696058513 | 398 | 4.5e-110 |
| WP\_063764077.1 | hypothetical\_protein | BGC0001348 | JBIR-100\_biosynthetic\_gene\_cluster | 32 | 36.9362047948 | 398 | 4.5e-110 |
| AEP40940.1 | polyketide\_synthase\_type\_I | BGC0000021 | Apoptolidin\_biosynthetic\_gene\_cluster | 32 | 36.6111336855 | 398 | 4.5e-110 |
| AAF71767.1 | NysJ | BGC0000115 | Nystatin\_biosynthetic\_gene\_cluster | 29 | 43.0719219829 | 398 | 5.9e-110 |
| ACY06288.1 | type\_I\_polyketide\_synthase | BGC0001042 | Sanglifehrin\_A\_biosynthetic\_gene\_cluster | 33 | 36.4892320195 | 398 | 5.9e-110 |
| AHA12079.1 | polyketide\_synthase\_type\_1 | BGC0001172 | Chlorizidine\_A\_biosynthetic\_gene\_cluster | 31 | 36.7330353515 | 398 | 5.9e-110 |
| AIW00670.1 | mellein\_synthase | BGC0001244 | (-)-Mellein\_biosynthetic\_gene\_cluster | 30 | 40.2275497765 | 398 | 7.7e-110 |
| AEH42473.1 | polyketide\_synthase | BGC0000032 | Calcimycin\_biosynthetic\_gene\_cluster | 32 | 35.3108492483 | 397 | 1e-109 |
| ABM21569.1 | CrpA | BGC0000975 | Cryptophycin\_biosynthetic\_gene\_cluster | 31 | 43.1125558716 | 397 | 1.3e-109 |
| AGI99496.1 | Type\_I\_polyketide\_synthase | BGC0001004 | Lobophorin\_biosynthetic\_gene\_cluster | 30 | 48.2730597318 | 396 | 1.7e-109 |
| ABJ97439.1 | MerC | BGC0001012 | Meridamycin\_biosynthetic\_gene\_cluster | 29 | 46.4851686306 | 396 | 1.7e-109 |
| AAX98190.1 | polyketide\_synthase\_type\_I | BGC0000052 | ECO-02301\_biosynthetic\_gene\_cluster | 31 | 38.236489232 | 396 | 2.3e-109 |
| BAG85030.1 | putative\_polyketide\_synthase | BGC0000086 | Lasalocid\_biosynthetic\_gene\_cluster | 32 | 37.1393742381 | 396 | 2.3e-109 |
| CAQ64690.1 | lasalocid\_modular\_polyketide\_synthase | BGC0000087 | Lasalocid\_biosynthetic\_gene\_cluster | 32 | 37.1393742381 | 396 | 2.3e-109 |
| BAF92601.1 | iterative\_type\_I\_PKS | BGC0000118 | Pactamycin\_biosynthetic\_gene\_cluster | 31 | 40.9995936611 | 396 | 2.9e-109 |
| ACJ24875.1 | 6-methylsalicylic\_acid\_synthase | BGC0000119 | Pactamycin\_biosynthetic\_gene\_cluster | 31 | 40.9995936611 | 396 | 2.9e-109 |
| AEU11005.1 | NpnA | BGC0001029 | Nostophycin\_biosynthetic\_gene\_cluster | 28 | 47.4197480699 | 395 | 5e-109 |
| BAH02271.1 | polyketide\_synthase | BGC0000126 | Pladienolide\_biosynthetic\_gene\_cluster | 32 | 37.3831775701 | 395 | 5e-109 |
| ACB46485.1 | polyketide\_synthase | BGC0000082 | Kijanimicin\_biosynthetic\_gene\_cluster | 32 | 37.3425436814 | 395 | 5e-109 |
| BAF85838.1 | modular\_polyketide\_synthase | BGC0000109 | Nemadectin\_biosynthetic\_gene\_cluster | 28 | 48.5574969525 | 394 | 6.6e-109 |
| AAD03048.1 | type\_I\_polyketide\_synthase | BGC0000041 | Coronafacic\_acid\_biosynthetic\_gene\_cluster | 32 | 37.0174725721 | 394 | 8.6e-109 |
| ABV83222.1 | CppJ | BGC0000116 | Nystatin-like\_Pseudonocardia\_polyene\_biosynthetic\_gene\_cluster | 32 | 35.8797236896 | 394 | 8.6e-109 |
| ALJ49910.1 | TlmH | BGC0001237 | Thiolactomycin\_biosynthetic\_gene\_cluster | 32 | 37.0174725721 | 394 | 1.1e-108 |
| ADU85988.1 | putative\_iterative\_type\_I\_polyketide\_synthase | BGC0000165 | Tiacumicin\_B\_biosynthetic\_gene\_cluster | 30 | 39.5367736692 | 393 | 1.5e-108 |
| ABC84471.1 | NigAVII | BGC0000114 | Nigericin\_biosynthetic\_gene\_cluster | 31 | 37.2206420154 | 393 | 1.5e-108 |
| AAP42867.1 | NanA7 | BGC0000105 | Nanchangmycin\_biosynthetic\_gene\_cluster | 32 | 36.9768386835 | 393 | 1.5e-108 |
| AGY62758.1 | EbeF | BGC0000051 | Ebelactone\_biosynthetic\_gene\_cluster | 28 | 45.7537586347 | 393 | 1.9e-108 |
| AAF86396.1 | FkbA | BGC0000994 | FK520\_biosynthetic\_gene\_cluster | 33 | 36.1235270215 | 393 | 1.9e-108 |
| AAG23264.1 | polyketide\_synthase\_loading\_and\_extender\_module\_1 | BGC0000148 | Spinosad\_biosynthetic\_gene\_cluster | 31 | 36.8143031288 | 393 | 1.9e-108 |
| AGI99494.1 | Type\_I\_polyketide\_synthase | BGC0001004 | Lobophorin\_biosynthetic\_gene\_cluster | 32 | 38.3990247867 | 392 | 3.3e-108 |
| BAQ21948.1 | putative\_type\_I\_polyketide\_synthase | BGC0001204 | Versipelostatin\_biosynthetic\_gene\_cluster | 32 | 37.5863470134 | 392 | 3.3e-108 |
| CBW54671.1 | polyketide\_synthase/non\_ribosomal\_peptide\_synthetase | BGC0000971 | Cinnabaramide\_biosynthetic\_gene\_cluster | 32 | 35.6359203576 | 392 | 3.3e-108 |
| AAM54076.1 | polyketide\_synthase | BGC0000020 | Ansamitocin\_biosynthetic\_gene\_cluster | 33 | 37.4238114588 | 392 | 4.2e-108 |
| CUI25727.1 | Polyketide\_synthase/Nonribosomal\_peptide\_synthase | BGC0001355 | U-68204\_biosynthetic\_gene\_cluster | 29 | 46.8508736286 | 392 | 4.2e-108 |
| AJW65408.1 | type\_I\_modular\_polyketide\_synthase | BGC0001195 | Nocardiopsin\_biosynthetic\_gene\_cluster | 32 | 37.2206420154 | 391 | 7.2e-108 |
| ADH04658.1 | TugB | BGC0001342 | Thuggacin\_biosynthetic\_gene\_cluster | 31 | 38.114587566 | 391 | 7.2e-108 |
| BAD38874.1 | polyketide\_synthase | BGC0000111 | Neocarzilin\_biosynthetic\_gene\_cluster | 33 | 35.7171881349 | 391 | 9.5e-108 |
| AHA38203.1 | GphJ | BGC0000069 | Gephyronic\_acid\_biosynthetic\_gene\_cluster | 33 | 37.0987403495 | 391 | 9.5e-108 |
| AJO72742.1 | Type\_I\_modular\_polyketide\_synthase | BGC0001381 | Brasilinolide\_biosynthetic\_gene\_cluster | 34 | 34.0918325884 | 390 | 1.2e-107 |
| AHB82062.1 | polyketide\_synthase | BGC0001231 | Microsclerodermins\_biosynthetic\_gene\_cluster | 29 | 46.688338074 | 390 | 1.2e-107 |
| AAK73513.1 | AmphB | BGC0000015 | Amphotericin\_biosynthetic\_gene\_cluster | 31 | 37.2206420154 | 390 | 1.2e-107 |
| AIG62146.1 | 6-methylsalicylic\_acid\_synthase | BGC0000120 | Patulin\_biosynthetic\_gene\_cluster | 31 | 36.5704997968 | 389 | 2.8e-107 |
| AJO72734.1 | Type\_I\_modular\_polyketide\_synthase | BGC0001381 | Brasilinolide\_biosynthetic\_gene\_cluster | 31 | 38.3177570093 | 389 | 3.6e-107 |
| BAE93722.1 | type\_I\_polyketide\_synthase | BGC0000164 | Tetronomycin\_biosynthetic\_gene\_cluster | 28 | 46.2413652987 | 388 | 4.7e-107 |
| BAQ25507.1 | type\_I\_polyketide\_synthase | BGC0001288 | Maklamicin\_biosynthetic\_gene\_cluster | 31 | 39.9024786672 | 388 | 4.7e-107 |
| AHB82053.1 | polyketide\_synthase | BGC0001019 | Microsclerodermins\_biosynthetic\_gene\_cluster | 31 | 37.0174725721 | 388 | 6.1e-107 |
| BAK64649.1 | polyketide\_synthase | BGC0000135 | Reveromycin\_biosynthetic\_gene\_cluster | 32 | 37.9114181227 | 387 | 1e-106 |
| ACB46487.1 | polyketide\_synthase | BGC0000082 | Kijanimicin\_biosynthetic\_gene\_cluster | 30 | 46.566436408 | 386 | 1.8e-106 |
| ABP53498.1 | beta-ketoacyl\_synthase | BGC0001041 | Salinosporamide\_biosynthetic\_gene\_cluster | 33 | 36.0828931329 | 386 | 2.3e-106 |
| CUI25702.1 | Tu3010\_Hybrid\_PKS-NRPS\_system | BGC0001352 | Thiotetronate\_Tu\_3010\_biosynthetic\_gene\_cluster | 29 | 44.7785453068 | 386 | 2.3e-106 |
| AAB66504.1 | tylactone\_synthase\_starter\_module\_and\_modules\_1\_&\_2 | BGC0000166 | Tylactone\_biosynthetic\_gene\_cluster | 31 | 37.3831775701 | 386 | 3e-106 |
| AAB66506.1 | tylactone\_synthase\_modules\_4\_&\_5 | BGC0000166 | Tylactone\_biosynthetic\_gene\_cluster | 32 | 37.3831775701 | 385 | 4e-106 |
| AFL48520.1 | laidlomycin\_polyketide\_synthase\_(module\_9) | BGC0000084 | Laidlomycin\_biosynthetic\_gene\_cluster | 31 | 36.3266964649 | 385 | 5.2e-106 |
| ABP73645.1 | SalA | BGC0000145 | Salinosporamide\_A\_biosynthetic\_gene\_cluster | 33 | 36.1235270215 | 385 | 5.2e-106 |
| CCA29203.1 | non-ribosomal\_peptide\_synthetase/polyketide\_synthase | BGC0000955 | Althiomycin\_biosynthetic\_gene\_cluster | 32 | 38.8053636733 | 383 | 1.5e-105 |
| AAP42855.1 | NanA1 | BGC0000105 | Nanchangmycin\_biosynthetic\_gene\_cluster | 32 | 38.8053636733 | 383 | 1.5e-105 |
| CAJ88175.1 | putative\_polyketide\_synthase\_B | BGC0000151 | Stambomycin\_biosynthetic\_gene\_cluster | 31 | 37.2612759041 | 383 | 2.6e-105 |
| ABY21541.1 | AngAIV | BGC0000018 | Angolamycin\_biosynthetic\_gene\_cluster | 31 | 36.4892320195 | 383 | 2.6e-105 |
| AAZ77693.1 | ChlA1 | BGC0000036 | Chlorothricin\_biosynthetic\_gene\_cluster | 31 | 37.7895164567 | 382 | 4.4e-105 |
| BAK64638.1 | polyketide\_synthase | BGC0000135 | Reveromycin\_biosynthetic\_gene\_cluster | 28 | 48.3136936205 | 381 | 9.8e-105 |
| ANF07288.1 | hrPKS | BGC0001340 | Byssochlamic\_acid\_biosynthetic\_gene\_cluster | 27 | 47.4197480699 | 381 | 9.8e-105 |
| BAC57029.1 | protomycinolide\_IV\_synthase\_2 | BGC0000102 | Mycinamicin\_biosynthetic\_gene\_cluster | 27 | 49.1263713937 | 381 | 9.8e-105 |
| CAD17792.1 | probable\_non\_ribosomal\_peptide\_synthetase\_protein | BGC0001363 | Ralsolamycin\_biosynthetic\_gene\_cluster | 32 | 36.5704997968 | 381 | 9.8e-105 |
| AAS98784.1 | JamM | BGC0001001 | Jamaicamide\_biosynthetic\_gene\_cluster | 32 | 34.498171475 | 380 | 1.7e-104 |
| ADH04640.1 | TgaB | BGC0001051 | Thuggacin\_biosynthetic\_gene\_cluster | 30 | 43.6001625356 | 380 | 1.7e-104 |
| AAO65801.1 | monensin\_polyketide\_synthase\_module\_9 | BGC0000100 | Monensin\_biosynthetic\_gene\_cluster | 32 | 36.8143031288 | 379 | 2.8e-104 |
| AAC38076.1 | polyketide\_synthase\_type\_I | BGC0000127 | Pyoluteorin\_biosynthetic\_gene\_cluster | 30 | 42.2998780983 | 379 | 3.7e-104 |
| AAP85336.1 | type\_I\_PKS | BGC0000233 | Hedamycin\_biosynthetic\_gene\_cluster | 33 | 37.0581064608 | 378 | 6.3e-104 |
| AJO72743.1 | Type\_I\_modular\_polyketide\_synthase | BGC0001381 | Brasilinolide\_biosynthetic\_gene\_cluster | 33 | 33.7261275904 | 378 | 8.3e-104 |
| BAF85844.1 | modular\_polyketide\_synthase | BGC0000109 | Nemadectin\_biosynthetic\_gene\_cluster | 31 | 37.4238114588 | 377 | 1.1e-103 |
| AHF22854.1 | MarL | BGC0000091 | Marineosin\_biosynthetic\_gene\_cluster | 31 | 35.6765542462 | 376 | 1.8e-103 |
| ACA99172.1 | Polyketide\_synthase | BGC0001160 | Nonadecene\_biosynthetic\_gene\_cluster | 31 | 36.1641609102 | 376 | 2.4e-103 |
| BAO66543.1 | type\_I\_polyketide\_synthase | BGC0000042 | Cremimycin\_biosynthetic\_gene\_cluster | 31 | 38.9272653393 | 374 | 7e-103 |
| AEK75504.1 | type\_1\_polyketide\_synthase | BGC0000001 | Abyssomicin\_biosynthetic\_gene\_cluster | 31 | 36.3673303535 | 371 | 1e-101 |
| AEE88283.1 | CurG | BGC0000976 | Curacin\_biosynthetic\_gene\_cluster | 31 | 34.498171475 | 370 | 1.3e-101 |
| AHB82065.1 | polyketide\_synthase | BGC0001231 | Microsclerodermins\_biosynthetic\_gene\_cluster | 36 | 26.655830963 | 370 | 1.3e-101 |
| AAT70102.1 | CurG | BGC0001165 | Curacin\_biosynthetic\_gene\_cluster | 31 | 34.498171475 | 370 | 1.3e-101 |
| CBD77746.1 | non-ribosomal\_peptide\_synthetase/polyketide\_synthase | BGC0000974 | Crocacin\_biosynthetic\_gene\_cluster | 31 | 42.3811458757 | 369 | 2.3e-101 |
| ACR50785.1 | polyketide\_synthase | BGC0000163 | Tetronasin\_biosynthetic\_gene\_cluster | 30 | 39.2929703373 | 369 | 2.3e-101 |
| AAB66508.1 | tylactone\_synthase\_module\_7 | BGC0000166 | Tylactone\_biosynthetic\_gene\_cluster | 33 | 34.620073141 | 369 | 3.9e-101 |
| AAX35547.1 | polyketide\_syntase\_2 | BGC0001275 | 6-methylsalicyclic\_acid\_biosynthetic\_gene\_cluster | 29 | 42.9906542056 | 368 | 5e-101 |
| ADH04682.1 | polyketide\_synthase | BGC0001344 | Tubulysin\_biosynthetic\_gene\_cluster | 32 | 36.9768386835 | 366 | 2.5e-100 |
| CAQ34929.1 | putative\_polyketide\_synthase | BGC0000986 | Dkxanthene\_biosynthetic\_gene\_cluster | 32 | 35.8390898009 | 364 | 1.2e-99 |
| AAR87760.2 | ZmaK | BGC0001059 | Zwittermycin\_A\_biosynthetic\_gene\_cluster | 32 | 36.1641609102 | 362 | 3.6e-99 |
| AHB82054.1 | polyketide\_synthase | BGC0001019 | Microsclerodermins\_biosynthetic\_gene\_cluster | 36 | 26.8183665177 | 362 | 4.7e-99 |
| AHD05619.1 | putative\_polyketide\_synthase\_subunit | BGC0001033 | Paenilamicin\_biosynthetic\_gene\_cluster | 31 | 37.2612759041 | 361 | 8e-99 |
| AHE80992.1 | PieA2 | BGC0001169 | Piericidin\_A1\_biosynthetic\_gene\_cluster | 30 | 36.2454286875 | 361 | 8e-99 |
| AFV52200.1 | polyketide\_synthase\_module | BGC0000081 | Kedarcidin\_biosynthetic\_gene\_cluster | 30 | 37.8707842341 | 361 | 1e-98 |
| CAE14173.1 |  | BGC0000383 | Luminmycin\_biosynthetic\_gene\_cluster | 29 | 46.1194636327 | 360 | 1.4e-98 |
| AGY62753.1 | EbeA | BGC0000051 | Ebelactone\_biosynthetic\_gene\_cluster | 32 | 33.8480292564 | 360 | 1.8e-98 |
| CAL80821.1 | sylD-like\_NRPS/PKS | BGC0000997 | Glidobactin\_biosynthetic\_gene\_cluster | 32 | 36.9768386835 | 359 | 3.1e-98 |
| AAS79463.1 | polyketide\_synthase\_subunit | BGC0000035 | Chalcomycin\_biosynthetic\_gene\_cluster | 30 | 37.8301503454 | 359 | 3.1e-98 |
| AHB82063.1 | polyketide\_synthase | BGC0001231 | Microsclerodermins\_biosynthetic\_gene\_cluster | 35 | 26.4526615197 | 358 | 6.8e-98 |
| AGC09485.1 | LobS2 | BGC0001183 | Lobophorin\_biosynthetic\_gene\_cluster | 29 | 48.2730597318 | 358 | 6.8e-98 |
| BAB69193.1 |  | BGC0000117 | Oligomycin\_biosynthetic\_gene\_cluster | 30 | 36.7330353515 | 358 | 8.9e-98 |
| AAP33839.2 | ochratoxin\_A\_polyketide\_synthase | BGC0001030 | Ochratoxin\_A\_biosynthetic\_gene\_cluster | 33 | 30.759853718 | 357 | 1.2e-97 |
| ADH04680.1 | hybrid\_polyketide\_synthase/non-ribosomal\_peptide\_synthetase | BGC0001344 | Tubulysin\_biosynthetic\_gene\_cluster | 32 | 38.0333197887 | 356 | 2e-97 |
| AAQ84144.1 | Plm4 | BGC0000123 | Phoslactomycin\_B\_biosynthetic\_gene\_cluster | 31 | 35.0264120276 | 356 | 2.6e-97 |
| AAZ94386.1 | modular\_polyketide\_synthase | BGC0000040 | Concanamycin\_A\_biosynthetic\_gene\_cluster | 31 | 37.3019097928 | 356 | 3.4e-97 |
| AHD05614.1 | putative\_non-ribosomal\_peptide\_ligase/\_polyketide\_synthase\_hybrid | BGC0001033 | Paenilamicin\_biosynthetic\_gene\_cluster | 31 | 36.8955709061 | 355 | 4.4e-97 |
| CAQ18839.1 | hybrid\_polyketide\_synthase/nonribosomal\_polypetide\_synthetase | BGC0000954 | Ajudazol\_biosynthetic\_gene\_cluster | 32 | 34.498171475 | 355 | 5.8e-97 |
| AAO62585.1 | peptide\_sythetase\_polyketide\_synthase\_fusion\_protein | BGC0001016 | Microcystin\_biosynthetic\_gene\_cluster | 30 | 37.2612759041 | 355 | 5.8e-97 |
| CAF05651.1 | TubF\_protein | BGC0001053 | Tubulysin\_biosynthetic\_gene\_cluster | 31 | 36.9768386835 | 354 | 7.5e-97 |
| CAD29795.1 | peptide\_synthetase | BGC0001015 | Microcystin\_biosynthetic\_gene\_cluster | 30 | 37.3019097928 | 354 | 1.3e-96 |
| ABY21542.1 | AngAV | BGC0000018 | Angolamycin\_biosynthetic\_gene\_cluster | 30 | 39.1304347826 | 351 | 1.1e-95 |
| ADZ24995.1 | non-ribosomal\_peptide\_synthase/polyketide\_synthase | BGC0000380 | Leupyrrin\_biosynthetic\_gene\_cluster | 29 | 47.2572125152 | 350 | 1.4e-95 |
| ABO15888.1 | polyketide\_synthase | BGC0000132 | Pyrrolomycin\_biosynthetic\_gene\_cluster | 33 | 31.5318976026 | 349 | 3.2e-95 |
| ANC94962.1 | AlmHV | BGC0001396 | Aldgamycin\_biosynthetic\_gene\_cluster | 30 | 38.0739536774 | 349 | 4.1e-95 |
| AHA38201.1 | GphH | BGC0000069 | Gephyronic\_acid\_biosynthetic\_gene\_cluster | 31 | 36.2860625762 | 347 | 9.2e-95 |
| BAE93725.1 | type\_I\_polyketide\_synthase | BGC0000164 | Tetronomycin\_biosynthetic\_gene\_cluster | 29 | 37.3831775701 | 347 | 9.2e-95 |
| AHA38200.1 | GphG | BGC0000069 | Gephyronic\_acid\_biosynthetic\_gene\_cluster | 25 | 67.5741568468 | 347 | 9.2e-95 |
| CAD15508.1 | probable\_polyketide\_synthase\_protein | BGC0001014 | Micacocidin\_biosynthetic\_gene\_cluster | 31 | 36.9768386835 | 347 | 1.2e-94 |
| AHB82070.1 | polyketide\_synthase | BGC0001231 | Microsclerodermins\_biosynthetic\_gene\_cluster | 31 | 36.8549370175 | 346 | 2e-94 |
| ABB52545.1 | putative\_type\_I\_polyketide\_synthase | BGC0000047 | Dihydrochalcomycin\_biosynthetic\_gene\_cluster | 30 | 38.0739536774 | 346 | 2e-94 |
| BAQ25482.1 | type\_I\_polyketide\_synthase | BGC0001288 | Maklamicin\_biosynthetic\_gene\_cluster | 35 | 28.4843559529 | 345 | 6e-94 |
| ADI24926.1 | VrtA | BGC0000168 | Viridicatumtoxin\_biosynthetic\_gene\_cluster | 31 | 36.2047947989 | 345 | 6e-94 |
| AAS98787.1 | JamP | BGC0001001 | Jamaicamide\_biosynthetic\_gene\_cluster | 32 | 36.8549370175 | 344 | 7.8e-94 |
| AAF00957.1 | McyG | BGC0001017 | Microcystin\_biosynthetic\_gene\_cluster | 30 | 37.5050792361 | 344 | 1.3e-93 |
| EAL89339.1 | polyketide\_synthase,\_putative | BGC0001403 | Trypacidin\_biosynthetic\_gene\_cluster | 30 | 37.7082486794 | 342 | 5e-93 |
| CCM44338.1 | putative\_polyketide\_synthase | BGC0001056 | Zeamine\_biosynthetic\_gene\_cluster | 32 | 33.9292970337 | 342 | 5e-93 |
| EAU38791.1 | hypothetical\_protein | BGC0000161 | Terrein\_biosynthetic\_gene\_cluster | 29 | 38.5209264527 | 341 | 6.6e-93 |
| EJK79843.1 | amino\_acid\_adenylation\_enzyme/thioester\_reductase\_family\_protein | BGC0000436 | Syringolin\_A\_biosynthetic\_gene\_cluster | 29 | 44.2909386428 | 341 | 8.6e-93 |
| ABB88534.1 | polyketide\_synthase\_type\_I | BGC0000049 | E-492\_/\_E-975\_biosynthetic\_gene\_cluster | 28 | 46.6477041853 | 340 | 1.5e-92 |
| ADA69241.1 | cis-AT\_polyketide\_synthase | BGC0001071 | Nosperin\_biosynthetic\_gene\_cluster | 33 | 34.132466477 | 337 | 1.6e-91 |
| ABI91466.1 | beta-ketoacyl\_synthase | BGC0001094 | Enacyloxin\_biosynthetic\_gene\_cluster | 29 | 36.9362047948 | 336 | 2.1e-91 |
| AEN83889.1 | AdaA | BGC0000156 | TAN-1612\_biosynthetic\_gene\_cluster | 26 | 48.5168630638 | 334 | 8e-91 |
| CBJ89764.1 | Polyketide\_synthase\_involved\_in\_xenocoumacin\_synthesis | BGC0001054 | Xenocoumacin\_biosynthetic\_gene\_cluster | 29 | 36.4892320195 | 332 | 5.2e-90 |
| EAL84397.1 | polyketide\_synthase,\_putative | BGC0001118 | Endocrocin\_biosynthetic\_gene\_cluster | 29 | 41.5684681024 | 330 | 1.5e-89 |
| AHB82052.1 | polyketide\_synthase | BGC0001019 | Microsclerodermins\_biosynthetic\_gene\_cluster | 33 | 26.9402681837 | 327 | 1.3e-88 |
| CBF90097.1 | hypothetical\_protein\_similar\_to\_polyketide\_synthase\_(Broad) | BGC0001271 | Emericellin\_biosynthetic\_gene\_cluster | 29 | 37.8301503454 | 327 | 1.3e-88 |
| CDG12864.1 | non-ribosomal\_peptide\_synthetase | BGC0001415 | Althiomycin\_biosynthetic\_gene\_cluster | 31 | 36.6111336855 | 326 | 2.2e-88 |
| AGO59040.1 | PtaA | BGC0000121 | Pestheic\_acid\_biosynthetic\_gene\_cluster | 28 | 38.1552214547 | 325 | 4.9e-88 |
| BAQ25481.1 | type\_I\_polyketide\_synthase | BGC0001288 | Maklamicin\_biosynthetic\_gene\_cluster | 34 | 28.8906948395 | 325 | 6.4e-88 |
| WP\_010639241.1 | beta-ketoacyl\_synthase | BGC0000958 | Antimycin\_biosynthetic\_gene\_cluster | 31 | 37.3831775701 | 324 | 8.3e-88 |
| AAZ94390.1 | modular\_polyketide\_synthase | BGC0000040 | Concanamycin\_A\_biosynthetic\_gene\_cluster | 29 | 38.5209264527 | 324 | 1.1e-87 |
| CAF05649.1 | TubD\_protein | BGC0001053 | Tubulysin\_biosynthetic\_gene\_cluster | 31 | 31.8569687119 | 321 | 7e-87 |
| AFD30954.1 | CrmA | BGC0000966 | Caerulomycin\_A\_biosynthetic\_gene\_cluster | 31 | 37.1800081268 | 321 | 1.2e-86 |
| AAZ95017.1 | polyketide\_synthase | BGC0000048 | Dothistromin\_biosynthetic\_gene\_cluster | 28 | 37.7488825681 | 321 | 1.2e-86 |
| CAO85898.1 | modular\_polyketide\_synthase\_NorC | BGC0000110 | Neoaureothin\_biosynthetic\_gene\_cluster | 34 | 26.9809020723 | 320 | 1.6e-86 |
| ADM79459.1 | PKS16\_protein | BGC0001266 | Grayanic\_acid\_biosynthetic\_gene\_cluster | 30 | 37.6676147907 | 319 | 2.7e-86 |
| ACD39770.1 | non-reducing\_polyketide\_synthase | BGC0000134 | Radicicol\_biosynthetic\_gene\_cluster | 29 | 36.9362047948 | 317 | 1.3e-85 |
| AKA54627.1 | PKS | BGC0001216 | Splenocin\_biosynthetic\_gene\_cluster | 30 | 37.6269809021 | 317 | 1.7e-85 |
| CAQ43079.1 | polyketide\_synthase | BGC0000970 | Chondrochloren\_biosynthetic\_gene\_cluster | 36 | 24.2177976432 | 315 | 5.1e-85 |
| AEP40925.1 | polyketide\_synthase\_type\_I | BGC0000021 | Apoptolidin\_biosynthetic\_gene\_cluster | 27 | 45.59122308 | 314 | 8.6e-85 |
| ABB90282.1 | polyketide\_synthase | BGC0001057 | Zearalenone\_biosynthetic\_gene\_cluster | 29 | 37.1800081268 | 314 | 1.1e-84 |
| BAD22832.1 | polyketide\_synthase | BGC0001265 | Melanin\_biosynthetic\_gene\_cluster | 30 | 37.0987403495 | 313 | 1.9e-84 |
| ABX37384.1 | Beta-ketoacyl\_synthase | BGC0000984 | Delftibactin\_biosynthetic\_gene\_cluster | 31 | 34.498171475 | 313 | 1.9e-84 |
| CAJ87591.1 | putative\_peptide/polyketide\_synthetase | BGC0001055 | Yersiniabactin\_biosynthetic\_gene\_cluster | 29 | 36.7330353515 | 313 | 2.5e-84 |
| ACR50795.1 | putative\_polyketide\_synthase | BGC0000163 | Tetronasin\_biosynthetic\_gene\_cluster | 33 | 26.0056887444 | 313 | 2.5e-84 |
| ABF87031.1 | non-ribosomal\_peptide\_synthetase/polyketide\_synthase | BGC0000393 | Myxoprincomide\_biosynthetic\_gene\_cluster | 30 | 37.1800081268 | 313 | 2.5e-84 |
| AGC95321.1 | CurS2 | BGC0000045 | Dehydrocurvularin\_biosynthetic\_gene\_cluster | 29 | 36.7330353515 | 312 | 4.3e-84 |
| AAS92537.1 | PKS1 | BGC0001044 | Sirodesmin\_biosynthetic\_gene\_cluster | 28 | 39.7805770012 | 312 | 4.3e-84 |
| CAA73127.1 | HMWP1\_protein | BGC0000467 | Yersiniabactin\_biosynthetic\_gene\_cluster | 29 | 36.7330353515 | 312 | 4.3e-84 |
| AAT69682.1 | polyketide\_synthase | BGC0001247 | Cercosporin\_biosynthetic\_gene\_cluster | 28 | 37.7895164567 | 312 | 4.3e-84 |
| CBJ89766.1 | Polyketide\_synthase\_involved\_in\_xenocoumacin\_synthesis | BGC0001054 | Xenocoumacin\_biosynthetic\_gene\_cluster | 31 | 33.4010564811 | 311 | 7.3e-84 |
| ACM79805.1 | ZmaA | BGC0001059 | Zwittermycin\_A\_biosynthetic\_gene\_cluster | 29 | 37.9520520114 | 310 | 2.1e-83 |
| CCE67070.1 | polyketide\_synthase | BGC0001242 | Fusarubin\_biosynthetic\_gene\_cluster | 29 | 37.7895164567 | 309 | 3.6e-83 |
| AAC49191.1 | putative\_polyketide\_synthase | BGC0000152 | Sterigmatocystin\_biosynthetic\_gene\_cluster | 29 | 36.7736692401 | 308 | 6.2e-83 |
| AEZ54376.1 | PieA3 | BGC0000124 | Piericidin\_A1\_biosynthetic\_gene\_cluster | 32 | 27.5091426249 | 308 | 8.1e-83 |
| CBF74114.1 | Conidial\_yellow\_pigment\_biosynthesis\_polyketide\_synthase\_(PKS)(EC\_2.3.1.-)\_[Source:UniProtKB/Swiss-Prot;Acc:Q03149] | BGC0000107 | Naphthopyrone\_biosynthetic\_gene\_cluster | 27 | 47.9479886225 | 308 | 8.1e-83 |
| ACH72912.1 | AflC | BGC0000011 | Aflatoxin/sterigmatocystin\_biosynthetic\_gene\_cluster | 27 | 37.0987403495 | 307 | 1.1e-82 |
| ABC34108.1 | JamP | BGC0000961 | Bactobolin\_biosynthetic\_gene\_cluster | 28 | 35.8390898009 | 307 | 1.1e-82 |
| EED53479.1 | polyketide\_synthase,\_putative | BGC0001304 | Aflavarin\_biosynthetic\_gene\_cluster | 29 | 37.3831775701 | 307 | 1.1e-82 |
| EGJ35088.1 | polyketide\_synthase\_module | BGC0001163 | 1-heptadecene\_biosynthetic\_gene\_cluster | 27 | 39.5367736692 | 307 | 1.4e-82 |
| CAQ34917.1 | polyketide\_synthase | BGC0000986 | Dkxanthene\_biosynthetic\_gene\_cluster | 38 | 20.5607476636 | 306 | 2.3e-82 |
| EGD99348.1 | polyketide\_synthase | BGC0001144 | Neosartoricin\_biosynthetic\_gene\_cluster | 28 | 36.9362047948 | 306 | 2.3e-82 |
| AHB82057.1 | polyketide\_synthase | BGC0001019 | Microsclerodermins\_biosynthetic\_gene\_cluster | 30 | 36.8955709061 | 304 | 8.9e-82 |
| AHB38509.1 | EpnH | BGC0000345 | Eponemycin\_biosynthetic\_gene\_cluster | 29 | 36.6111336855 | 302 | 3.4e-81 |
| ERM18799.1 | polyketide\_synthase | BGC0000172 | Basiliskamides\_biosynthetic\_gene\_cluster | 37 | 21.4546932141 | 300 | 1.3e-80 |
| AHH34186.1 | polyketide\_synthase | BGC0001161 | 1-heptadecene\_biosynthetic\_gene\_cluster | 27 | 39.3742381146 | 300 | 1.7e-80 |
| AFP87524.1 | type\_I\_polyketide\_synthase | BGC0001159 | Marinopyrrole\_biosynthetic\_gene\_cluster | 33 | 26.8183665177 | 300 | 1.7e-80 |
| CCT67991.1 | bikaverin\_cluster-polyketide\_synthase | BGC0000030 | Bikaverin\_biosynthetic\_gene\_cluster | 28 | 37.2612759041 | 299 | 4.9e-80 |
| CBF79143.1 | polyketide\_synthase,\_putative\_(JCVI) | BGC0000013 | Alternariol\_biosynthetic\_gene\_cluster | 27 | 39.1304347826 | 299 | 4.9e-80 |
| AGY62756.1 | EbeD | BGC0000051 | Ebelactone\_biosynthetic\_gene\_cluster | 33 | 23.2425843153 | 297 | 1.1e-79 |
| AAO65790.1 | ketosynthase-like\_protein | BGC0000100 | Monensin\_biosynthetic\_gene\_cluster | 32 | 28.3624542869 | 297 | 1.1e-79 |
| CCH26295.1 | putative\_polyketide\_synthase | BGC0001158\_c6 | Bikaverin\_biosynthetic\_gene\_cluster | 28 | 38.2771231207 | 297 | 1.4e-79 |
| AHV78253.1 | ResS2 | BGC0001246 | Trans-resorcylide\_biosynthetic\_gene\_cluster | 28 | 38.114587566 | 295 | 4.1e-79 |
| AHV78247.1 | LasS2 | BGC0001245 | Lasiodiplodin\_biosynthetic\_gene\_cluster | 29 | 36.2860625762 | 293 | 2.1e-78 |
| ADI59534.1 | CorL | BGC0001091 | Corallopyronin\_biosynthetic\_gene\_cluster | 37 | 20.2763104429 | 293 | 2.1e-78 |
| AFX60336.1 | polyketide\_synthase | BGC0001032 | Oocydin\_A\_biosynthetic\_gene\_cluster | 37 | 21.1296221048 | 293 | 2.1e-78 |
| CAD70195.1 | non-ribosomal\_peptide\_synthetase | BGC0001047 | Syringolin\_A\_biosynthetic\_gene\_cluster | 27 | 44.2909386428 | 292 | 6e-78 |
| CBW45741.1 | hybrid\_PKS/NRPS\_(PKSI) | BGC0000952\_c3 | Pristinamycin\_biosynthetic\_gene\_cluster | 36 | 21.1296221048 | 291 | 7.8e-78 |
| ABI91469.1 | beta-ketoacyl\_synthase | BGC0001094 | Enacyloxin\_biosynthetic\_gene\_cluster | 32 | 26.0869565217 | 291 | 7.8e-78 |
| AKA59448.1 | polyketide\_synthase | BGC0001203 | Clarexpoxcin\_biosynthetic\_gene\_cluster | 31 | 35.2702153596 | 291 | 7.8e-78 |
| ADF88262.1 | mixed\_nonribosomal\_peptide\_synthetase/\_polyketide\_synthase | BGC0000979 | Cylindrospermopsin\_biosynthetic\_gene\_cluster | 33 | 27.021535961 | 290 | 2.3e-77 |
| ADF88265.1 | mixed\_nonribosomal\_peptide\_synthetase/\_polyketide\_synthase | BGC0000980 | Cylindrospermopsin\_biosynthetic\_gene\_cluster | 33 | 27.021535961 | 290 | 2.3e-77 |
| CAE14178.1 |  | BGC0001180 | Isopropylstilbene\_biosynthetic\_gene\_cluster | 36 | 20.6826493295 | 289 | 3e-77 |
| ACY01401.1 | AT-less\_polyketide\_synthase | BGC0000083 | Lactimidomycin\_biosynthetic\_gene\_cluster | 36 | 21.2921576595 | 289 | 5.1e-77 |
| ABF89568.1 | polyketide\_synthase | BGC0001025 | Myxovirescin\_biosynthetic\_gene\_cluster | 36 | 20.7232832182 | 289 | 5.1e-77 |
| ADI59533.1 | CorK | BGC0001091 | Corallopyronin\_biosynthetic\_gene\_cluster | 34 | 21.4140593255 | 287 | 1.1e-76 |
| ADN68478.1 | SorC | BGC0000184 | Sorangicin\_biosynthetic\_gene\_cluster | 38 | 20.7232832182 | 287 | 1.1e-76 |
| AFL48521.1 | beta-ketosynthase | BGC0000084 | Laidlomycin\_biosynthetic\_gene\_cluster | 32 | 26.6151970744 | 287 | 1.9e-76 |
| AFX60313.1 | polyketide\_synthase | BGC0001031 | Oocydin\_A\_biosynthetic\_gene\_cluster | 37 | 21.1296221048 | 287 | 1.9e-76 |
| ACD39753.1 | non-reducing\_polyketide\_synthase | BGC0000076 | Hypothemycin\_biosynthetic\_gene\_cluster | 28 | 36.5704997968 | 286 | 3.3e-76 |
| ACD39762.1 | non-reducing\_polyketide\_synthase | BGC0000077 | Hypothemycin\_biosynthetic\_gene\_cluster | 28 | 36.5704997968 | 286 | 3.3e-76 |
| ABF92489.1 | mixed\_type\_I\_polyketide\_synthase\_-\_peptide\_synthetase | BGC0001025 | Myxovirescin\_biosynthetic\_gene\_cluster | 34 | 21.6172287688 | 286 | 3.3e-76 |
| ADI59531.1 | CorI | BGC0001091 | Corallopyronin\_biosynthetic\_gene\_cluster | 34 | 21.5765948801 | 285 | 7.3e-76 |
| ADY00130.1 | polyketide\_synthase | BGC0000104 | Mycophenolic\_acid\_biosynthetic\_gene\_cluster | 28 | 37.0987403495 | 284 | 1.2e-75 |
| ABS90475.1 | PKS | BGC0001106 | Oxazolomycin\_biosynthetic\_gene\_cluster | 36 | 20.9670865502 | 284 | 1.2e-75 |
| CCA89328.1 | mixed\_trans-AT\_type\_I\_polyketide\_synthase/nonribosomal\_peptide\_synthetase | BGC0001111 | Rhizopodins\_biosynthetic\_gene\_cluster | 36 | 20.8451848842 | 284 | 1.6e-75 |
| CDM36726.1 | Beta-ketoacyl\_synthase | BGC0001360 | Mycophenolic\_acid\_biosynthetic\_gene\_cluster | 28 | 35.7171881349 | 283 | 2.1e-75 |
| AFX60309.1 | polyketide\_synthase | BGC0001031 | Oocydin\_A\_biosynthetic\_gene\_cluster | 36 | 21.0483543275 | 283 | 2.8e-75 |
| ALD83688.1 | tAT\_polyketide\_synthase | BGC0001300 | Anthracimycin\_biosynthetic\_gene\_cluster | 36 | 20.4388459976 | 283 | 2.8e-75 |
| AKA59437.1 | polyketide\_synthase | BGC0001202 | Landepoxcin\_biosynthetic\_gene\_cluster | 30 | 33.2385209265 | 282 | 3.6e-75 |
| AFX60332.1 | polyketide\_synthase | BGC0001032 | Oocydin\_A\_biosynthetic\_gene\_cluster | 36 | 20.5201137749 | 282 | 4.7e-75 |
| AAD38786.1 | polyketide\_synthase | BGC0001257 | 1,3,6,8-tetrahydroxynaphthalene\_biosynthetic\_gene\_cluster | 27 | 37.0987403495 | 280 | 1.8e-74 |
| CTQ34881.1 | AtcD;\_polyketide\_synthase,\_modules\_1-4 | BGC0001301 | Anthracimycin\_biosynthetic\_gene\_cluster | 36 | 20.5607476636 | 280 | 1.8e-74 |
| ABC36687.1 | polyketide\_synthase | BGC0000964 | Burkholdac\_biosynthetic\_gene\_cluster | 37 | 21.2515237708 | 280 | 1.8e-74 |
| ABC36203.1 | thiotemplate\_mechanism\_natural\_product\_synthetase | BGC0001102 | Malleilactone\_biosynthetic\_gene\_cluster | 28 | 38.1958553434 | 280 | 2.4e-74 |
| CUW01174.1 | Type\_I\_polyketide\_synthase | BGC0001359 | PM100117\_/\_PM100118\_biosynthetic\_gene\_cluster | 28 | 34.9451442503 | 279 | 3.1e-74 |
| AAS47564.1 | mixed\_type\_I\_polyketide\_synthase/nonribosomal\_peptide\_synthetase | BGC0001108 | Pederin\_biosynthetic\_gene\_cluster | 36 | 21.0889882162 | 279 | 4e-74 |
| ctg1\_orf6 |  | BGC0001109 | Pederin\_biosynthetic\_gene\_cluster | 36 | 21.0889882162 | 279 | 4e-74 |
| AAY89050.1 | polyketide\_synthase | BGC0001069 | Chivosazole\_biosynthetic\_gene\_cluster | 35 | 21.4953271028 | 279 | 4e-74 |
| AMH40421.1 | PKS | BGC0001350 | Phormidolide\_biosynthetic\_gene\_cluster | 35 | 20.6420154409 | 277 | 1.2e-73 |
| AMH40423.1 | PKS | BGC0001350 | Phormidolide\_biosynthetic\_gene\_cluster | 35 | 21.2108898822 | 277 | 1.5e-73 |
| CBW45748.1 | hybrid\_PKS/NRPS\_(PKSI) | BGC0000952\_c3 | Pristinamycin\_biosynthetic\_gene\_cluster | 36 | 22.3486387647 | 277 | 2e-73 |
| ADN68477.1 | SorB | BGC0000184 | Sorangicin\_biosynthetic\_gene\_cluster | 35 | 20.9670865502 | 276 | 2.6e-73 |
| AMH40443.1 | PKS | BGC0001350 | Phormidolide\_biosynthetic\_gene\_cluster | 35 | 20.4388459976 | 276 | 2.6e-73 |
| AAV97870.1 | OnnB | BGC0001105 | Onnamide\_biosynthetic\_gene\_cluster | 36 | 20.9670865502 | 276 | 3.4e-73 |
| AEC04363.1 | polyketide\_synthase | BGC0000178 | Elansolid\_biosynthetic\_gene\_cluster | 34 | 20.4794798862 | 276 | 3.4e-73 |
| ALD83702.1 | tAT\_polyketide\_synthase | BGC0001299 | Chlorotonil\_biosynthetic\_gene\_cluster | 37 | 21.4953271028 | 276 | 3.4e-73 |
| AAN59953.1 | polyketide\_synthase\_1 | BGC0001258 | 1,8-dihydroxynaphthalene\_biosynthetic\_gene\_cluster | 26 | 40.1869158879 | 275 | 4.4e-73 |
| ABI91465.1 | beta-ketoacyl\_synthase | BGC0001094 | Enacyloxin\_biosynthetic\_gene\_cluster | 36 | 20.1137748883 | 275 | 4.4e-73 |
| ABS90470.1 | NRPS/PKS | BGC0001106 | Oxazolomycin\_biosynthetic\_gene\_cluster | 35 | 21.7797643234 | 275 | 4.4e-73 |
| CCP20050.1 | divL3\_protein | BGC0001119 | Divergolide\_biosynthetic\_gene\_cluster | 34 | 20.9264526615 | 275 | 5.8e-73 |
| BAF50727.1 | hybrid\_polyketide\_synthase-non\_ribosomal\_peptide\_synthetase | BGC0001116 | Virginiamycin\_biosynthetic\_gene\_cluster | 36 | 20.9670865502 | 275 | 5.8e-73 |
| AAY32964.1 | DszA | BGC0001093 | Disorazole\_biosynthetic\_gene\_cluster | 35 | 20.4794798862 | 274 | 9.9e-73 |
| ALD83703.1 | tAT\_polyketide\_synthase | BGC0001299 | Chlorotonil\_biosynthetic\_gene\_cluster | 36 | 20.3575782202 | 274 | 1.7e-72 |
| CBF80428.1 | polyketide\_synthase,\_putative\_(JCVI) | BGC0000680 | Austinol/dehydroaustinol,\_locus\_1\_biosynthetic\_gene\_cluster | 25 | 47.7854530679 | 273 | 2.2e-72 |
| CAG23957.2 | NRPS/PKS\_protein | BGC0001089 | Bacillaene\_biosynthetic\_gene\_cluster | 34 | 22.3892726534 | 273 | 2.9e-72 |
| AMH40422.1 | PKS | BGC0001350 | Phormidolide\_biosynthetic\_gene\_cluster | 35 | 20.5201137749 | 273 | 2.9e-72 |
| BAE93740.1 | type\_I\_polyketide\_synthase-related\_protein | BGC0000164 | Tetronomycin\_biosynthetic\_gene\_cluster | 36 | 21.8610321008 | 272 | 3.8e-72 |
| AGN74892.1 | nonribosomal\_peptide\_synthetase/polyketide\_synthase\_hybrid\_protein | BGC0000459 | Griseoviridin\_/\_viridogrisein\_biosynthetic\_gene\_cluster | 33 | 23.4863876473 | 272 | 4.9e-72 |
| BAD38875.1 | polyketide\_synthase | BGC0000111 | Neocarzilin\_biosynthetic\_gene\_cluster | 35 | 21.3327915482 | 272 | 4.9e-72 |
| CAL69890.1 | RhiC\_protein | BGC0001112 | Rhizoxins\_biosynthetic\_gene\_cluster | 35 | 21.3327915482 | 272 | 6.4e-72 |
| EHA28237.1 | hypothetical\_protein | BGC0001143 | Azanigerone\_biosynthetic\_gene\_cluster | 27 | 34.4169036977 | 272 | 6.4e-72 |
| ALD83686.1 | tAT\_polyketide\_synthase | BGC0001300 | Anthracimycin\_biosynthetic\_gene\_cluster | 35 | 20.4794798862 | 271 | 8.4e-72 |
| AAN85523.1 | polyketide\_synthase | BGC0001101 | Leinamycin\_biosynthetic\_gene\_cluster | 34 | 21.9016659894 | 271 | 1.1e-71 |
| CTQ34882.1 | AtcE;\_polyketide\_synthase,\_modules\_5-7 | BGC0001301 | Anthracimycin\_biosynthetic\_gene\_cluster | 34 | 22.4299065421 | 271 | 1.1e-71 |
| AKQ22696.1 | malonyl\_CoA-acyl\_carrier\_protein\_transacylase | BGC0001186 | Misakinolide\_biosynthetic\_gene\_cluster | 34 | 21.3327915482 | 270 | 1.4e-71 |
| AGN71604.1 | conidial\_yellow\_pigment\_biosynthesis\_polyketide\_synthase | BGC0000027 | Azaphilone\_biosynthetic\_gene\_cluster | 27 | 34.5794392523 | 270 | 1.4e-71 |
| ADA82581.1 | trans-AT\_polyketide\_synthase | BGC0001110 | Psymberin\_biosynthetic\_gene\_cluster | 36 | 20.0731409996 | 270 | 1.4e-71 |
| CCA89326.1 | mixed\_trans-AT\_type\_I\_polyketide\_synthase/nonribosomal\_peptide\_synthetase | BGC0001111 | Rhizopodins\_biosynthetic\_gene\_cluster | 33 | 21.0889882162 | 270 | 1.9e-71 |
| CAE52339.1 | Polyketide\_non-ribosomal\_peptide\_synthase | BGC0001088 | Albicidin\_biosynthetic\_gene\_cluster | 35 | 21.1296221048 | 270 | 1.9e-71 |
| AFO59866.1 | ChxE | BGC0000175 | Cycloheximide\_/\_actiphenol\_biosynthetic\_gene\_cluster | 34 | 20.7639171069 | 270 | 2.4e-71 |
| AKQ22698.1 | malonyl\_CoA-acyl\_carrier\_protein\_transacylase | BGC0001186 | Misakinolide\_biosynthetic\_gene\_cluster | 34 | 20.7232832182 | 270 | 2.4e-71 |
| AIC32694.1 | FR9GH | BGC0001113 | Spliceostatin\_/\_FR901464\_biosynthetic\_gene\_cluster | 34 | 21.2108898822 | 270 | 2.4e-71 |
| ADH01489.1 | type\_I\_polyketide\_synthase | BGC0000995 | FR901464\_biosynthetic\_gene\_cluster | 34 | 21.2108898822 | 270 | 2.4e-71 |
| CTQ34883.1 | AtcF;\_polyketide\_synthase,\_modules\_8-10 | BGC0001301 | Anthracimycin\_biosynthetic\_gene\_cluster | 35 | 20.6420154409 | 269 | 3.2e-71 |
| ABC34675.1 | polyketide\_synthase,\_putative | BGC0000186 | Thailandamide\_biosynthetic\_gene\_cluster | 36 | 20.7232832182 | 269 | 4.2e-71 |
| ADH01487.1 | polyketide\_synthase | BGC0000995 | FR901464\_biosynthetic\_gene\_cluster | 35 | 20.4388459976 | 269 | 5.4e-71 |
| AIC32693.1 | FR9DEF | BGC0001113 | Spliceostatin\_/\_FR901464\_biosynthetic\_gene\_cluster | 35 | 20.4388459976 | 269 | 5.4e-71 |
| BAV19379.1 | polyketide\_synthase | BGC0001390 | LL-Z1272beta\_biosynthetic\_gene\_cluster | 28 | 31.125558716 | 268 | 7.1e-71 |
| AGN11882.1 | TstGH | BGC0001114 | Thailanstatin\_biosynthetic\_gene\_cluster | 33 | 22.1861032101 | 268 | 7.1e-71 |
| AKQ22699.1 | malonyl\_CoA-acyl\_carrier\_protein\_transacylase | BGC0001186 | Misakinolide\_biosynthetic\_gene\_cluster | 35 | 21.9829337668 | 268 | 9.2e-71 |
| ABC35522.1 | thiotemplate\_mechanism\_natural\_product\_synthetase | BGC0000186 | Thailandamide\_biosynthetic\_gene\_cluster | 32 | 29.2970337261 | 267 | 1.2e-70 |
| AFX60311.1 | polyketide\_synthase | BGC0001031 | Oocydin\_A\_biosynthetic\_gene\_cluster | 34 | 21.3734254368 | 267 | 1.6e-70 |
| ADN68476.1 | SorA | BGC0000184 | Sorangicin\_biosynthetic\_gene\_cluster | 33 | 23.0800487607 | 266 | 2.7e-70 |
| AGN11881.1 | TstDEF | BGC0001114 | Thailanstatin\_biosynthetic\_gene\_cluster | 34 | 20.8045509955 | 266 | 3.5e-70 |
| AAY32965.1 | DszB | BGC0001093 | Disorazole\_biosynthetic\_gene\_cluster | 34 | 22.3486387647 | 266 | 3.5e-70 |
| ADD82939.1 | Bat1 | BGC0001099 | Kalimantacin\_/\_batumin\_biosynthetic\_gene\_cluster | 36 | 20.7232832182 | 265 | 4.6e-70 |
| ABF85931.1 | non-ribosomal\_peptide\_synthase/polyketide\_synthase\_Ta1 | BGC0001025 | Myxovirescin\_biosynthetic\_gene\_cluster | 34 | 21.4546932141 | 265 | 6e-70 |
| ALD83704.1 | tAT\_polyketide\_synthase | BGC0001299 | Chlorotonil\_biosynthetic\_gene\_cluster | 34 | 21.1296221048 | 265 | 6e-70 |
| ACY01391.1 | AT-less\_polyketide\_synthase | BGC0000177 | Dorrigocin\_/\_migrastatin\_biosynthetic\_gene\_cluster | 34 | 21.8610321008 | 265 | 6e-70 |
| CUI25673.1 | Thiolactomycin\_Polyketide\_synthase | BGC0001353 | Thiolactomycin\_biosynthetic\_gene\_cluster | 34 | 20.0731409996 | 265 | 7.8e-70 |
| AKQ22697.1 | malonyl\_CoA-acyl\_carrier\_protein\_transacylase | BGC0001186 | Misakinolide\_biosynthetic\_gene\_cluster | 33 | 23.7708248679 | 264 | 1e-69 |
| CAN89631.1 | putative\_polyketide\_synthase | BGC0001070 | Kirromycin\_biosynthetic\_gene\_cluster | 35 | 20.6826493295 | 264 | 1.7e-69 |
| BAD08358.1 | polyketide\_synthase\_modules\_4 | BGC0000167 | Vicenistatin\_biosynthetic\_gene\_cluster | 28 | 33.6042259244 | 264 | 1.7e-69 |
| ABC33986.1 | polyketide\_synthase,\_putative | BGC0000186 | Thailandamide\_biosynthetic\_gene\_cluster | 33 | 21.9829337668 | 264 | 1.7e-69 |
| ADD82941.1 | Bat3 | BGC0001099 | Kalimantacin\_/\_batumin\_biosynthetic\_gene\_cluster | 35 | 20.8858187729 | 263 | 2.3e-69 |
| ACY01390.1 | AT-less\_polyketide\_synthase | BGC0000177 | Dorrigocin\_/\_migrastatin\_biosynthetic\_gene\_cluster | 34 | 21.9422998781 | 263 | 2.3e-69 |
| AEC04362.1 | polyketide\_synthase | BGC0000178 | Elansolid\_biosynthetic\_gene\_cluster | 32 | 22.8362454287 | 263 | 2.3e-69 |
| CAO98848.1 | polyketide\_synthase\_AufE | BGC0000023 | Aurafuron\_biosynthetic\_gene\_cluster | 29 | 28.3624542869 | 263 | 2.3e-69 |
| AAV97877.1 | OnnI | BGC0001105 | Onnamide\_biosynthetic\_gene\_cluster | 35 | 21.1296221048 | 263 | 3e-69 |
| CCC21123.1 | type-I\_polyketide\_synthases | BGC0000171 | 9-methylstreptimidone\_biosynthetic\_gene\_cluster | 34 | 20.8451848842 | 263 | 3e-69 |
| ADN68479.1 | SorD | BGC0000184 | Sorangicin\_biosynthetic\_gene\_cluster | 35 | 21.1702559935 | 263 | 3e-69 |
| AAP42872.1 | NanA9 | BGC0000105 | Nanchangmycin\_biosynthetic\_gene\_cluster | 31 | 27.1028037383 | 262 | 3.9e-69 |
| ADA69237.1 | trans-AT\_polyketide\_synthase | BGC0001071 | Nosperin\_biosynthetic\_gene\_cluster | 35 | 21.2921576595 | 262 | 3.9e-69 |
| AEH42487.1 | polyketide\_synthase | BGC0000032 | Calcimycin\_biosynthetic\_gene\_cluster | 26 | 42.2592442097 | 261 | 1.1e-68 |
| AEH59109.1 | keto-hydroxyglutarate-aldolase/polyketide\_synthase | BGC0000385 | Lysobactin\_biosynthetic\_gene\_cluster | 36 | 20.4794798862 | 261 | 1.1e-68 |
| ACR13997.1 | modular\_polyketide\_synthase,\_type\_I\_PKS | BGC0000185 | Tartrolon\_biosynthetic\_gene\_cluster | 34 | 21.0889882162 | 260 | 1.5e-68 |
| BAC76474.1 | type\_I\_polyketide\_synthase\_LkcC | BGC0001100 | Lankacidin\_biosynthetic\_gene\_cluster | 33 | 20.1544087769 | 260 | 1.9e-68 |
| BAC76476.1 | multifunctional\_polyketide-peptide\_synthase\_LkcA | BGC0001100 | Lankacidin\_biosynthetic\_gene\_cluster | 34 | 20.4794798862 | 260 | 2.5e-68 |
| AEC04357.1 | polyketide\_synthase | BGC0000178 | Elansolid\_biosynthetic\_gene\_cluster | 34 | 21.6578626575 | 260 | 2.5e-68 |
| AEC04361.1 | polyketide\_synthase | BGC0000178 | Elansolid\_biosynthetic\_gene\_cluster | 32 | 23.6082893133 | 260 | 2.5e-68 |
| AAG23265.1 | polyketide\_synthase\_extender\_module\_2 | BGC0000148 | Spinosad\_biosynthetic\_gene\_cluster | 29 | 28.1999187322 | 259 | 3.3e-68 |
| CAG23983.1 | polyketide\_synthase\_type\_I | BGC0000176 | Difficidin\_biosynthetic\_gene\_cluster | 34 | 21.1702559935 | 259 | 3.3e-68 |
| ACY01400.1 | AT-less\_polyketide\_synthase | BGC0000083 | Lactimidomycin\_biosynthetic\_gene\_cluster | 33 | 21.8203982121 | 259 | 4.3e-68 |
| ADI59532.1 | CorJ | BGC0001091 | Corallopyronin\_biosynthetic\_gene\_cluster | 35 | 20.5201137749 | 258 | 7.3e-68 |
| ERM18798.1 | polyketide\_synthase | BGC0000172 | Basiliskamides\_biosynthetic\_gene\_cluster | 34 | 22.1454693214 | 258 | 9.6e-68 |
| AFN27480.1 | pks\_BonA | BGC0000173 | Bongkrekic\_acid\_biosynthetic\_gene\_cluster | 32 | 23.5676554246 | 258 | 9.6e-68 |
| AIU36103.1 | LglD | BGC0000180 | Legioliulin\_biosynthetic\_gene\_cluster | 34 | 20.7639171069 | 258 | 9.6e-68 |
| CBK62724.1 |  | BGC0001115 | Thiomarinol\_biosynthetic\_gene\_cluster | 34 | 20.5201137749 | 258 | 9.6e-68 |
| ERM18797.1 | polyketide\_synthase | BGC0000172 | Basiliskamides\_biosynthetic\_gene\_cluster | 34 | 21.0077204388 | 257 | 1.3e-67 |
| CAN93347.1 | Polyketide\_synthase | BGC0000179 | Etnangien\_biosynthetic\_gene\_cluster | 34 | 21.8610321008 | 257 | 1.3e-67 |
| ABM63528.1 | BryC | BGC0000174 | Bryostatin\_biosynthetic\_gene\_cluster | 33 | 21.0483543275 | 257 | 1.6e-67 |
| AAY89052.1 | polyketide\_synthase | BGC0001069 | Chivosazole\_biosynthetic\_gene\_cluster | 34 | 20.2763104429 | 257 | 2.1e-67 |
| AGN74894.1 | nonribosomal\_peptide\_synthetase/polyketide\_synthase\_hybrid\_protein | BGC0000459 | Griseoviridin\_/\_viridogrisein\_biosynthetic\_gene\_cluster | 33 | 21.4140593255 | 257 | 2.1e-67 |
| CBK62733.1 |  | BGC0001115 | Thiomarinol\_biosynthetic\_gene\_cluster | 34 | 20.9264526615 | 256 | 2.8e-67 |
| AAM12909.2 | MmpA | BGC0000182 | Mupirocin\_biosynthetic\_gene\_cluster | 34 | 20.8451848842 | 256 | 2.8e-67 |
| CAL69893.1 | RhiE\_protein | BGC0001112 | Rhizoxins\_biosynthetic\_gene\_cluster | 32 | 21.9829337668 | 256 | 3.6e-67 |
| BAP05594.1 | CalF | BGC0000967 | Calyculin\_biosynthetic\_gene\_cluster | 34 | 20.6420154409 | 255 | 4.8e-67 |
| ADN68482.1 | SorG | BGC0000184 | Sorangicin\_biosynthetic\_gene\_cluster | 36 | 20.6013815522 | 255 | 8.1e-67 |
| CUI25726.1 | Polyketide\_synthase | BGC0001355 | U-68204\_biosynthetic\_gene\_cluster | 34 | 20.0325071109 | 254 | 1.1e-66 |
| CAG23977.1 | polyketide\_synthase\_type\_I | BGC0000176 | Difficidin\_biosynthetic\_gene\_cluster | 32 | 24.2177976432 | 254 | 1.1e-66 |
| AAF26923.1 | polyketide\_synthase | BGC0000988 | Epothilone\_biosynthetic\_gene\_cluster | 28 | 28.8500609508 | 254 | 1.1e-66 |
| ABB86408.1 | GelA | BGC0000067 | Geldanamycin\_biosynthetic\_gene\_cluster | 27 | 31.8976026006 | 254 | 1.4e-66 |
| CAL69891.1 | RhiD\_protein | BGC0001112 | Rhizoxins\_biosynthetic\_gene\_cluster | 32 | 21.9422998781 | 253 | 2.4e-66 |
| ABS90471.1 | PKS\_type\_I | BGC0001106 | Oxazolomycin\_biosynthetic\_gene\_cluster | 33 | 22.0235676554 | 253 | 2.4e-66 |
| ADD82940.1 | Bat2 | BGC0001099 | Kalimantacin\_/\_batumin\_biosynthetic\_gene\_cluster | 34 | 20.7232832182 | 253 | 2.4e-66 |
| AAY89053.1 | polyketide\_synthase | BGC0001069 | Chivosazole\_biosynthetic\_gene\_cluster | 34 | 21.8203982121 | 253 | 2.4e-66 |
| AAF62885.1 | EpoF | BGC0000991 | Epothilone\_biosynthetic\_gene\_cluster | 28 | 32.6290125965 | 253 | 3.1e-66 |
| ABF88102.1 | polyketide\_synthase | BGC0001025 | Myxovirescin\_biosynthetic\_gene\_cluster | 33 | 21.4953271028 | 252 | 4e-66 |
| ADB12493.1 | EpoF | BGC0000990 | Epothilone\_biosynthetic\_gene\_cluster | 28 | 28.8500609508 | 252 | 5.3e-66 |
| CAN93351.1 | polyketide\_synthase | BGC0000179 | Etnangien\_biosynthetic\_gene\_cluster | 35 | 20.4388459976 | 252 | 6.9e-66 |
| ABM63527.1 | BryB | BGC0000174 | Bryostatin\_biosynthetic\_gene\_cluster | 33 | 21.2108898822 | 252 | 6.9e-66 |
| CAJ88176.1 | putative\_polyketide\_synthase\_B | BGC0000151 | Stambomycin\_biosynthetic\_gene\_cluster | 30 | 20.6420154409 | 252 | 6.9e-66 |
| AAO06916.1 | GdmAI | BGC0000066 | Geldanamycin\_biosynthetic\_gene\_cluster | 27 | 31.9788703779 | 251 | 9e-66 |
| ACB46197.1 | polyketide\_synthase | BGC0000989 | Epothilone\_biosynthetic\_gene\_cluster | 28 | 32.6696464852 | 251 | 9e-66 |
| BAP05593.1 | CalE | BGC0000967 | Calyculin\_biosynthetic\_gene\_cluster | 32 | 24.3803331979 | 251 | 9e-66 |
| AFX60334.1 | polyketide\_synthase | BGC0001032 | Oocydin\_A\_biosynthetic\_gene\_cluster | 33 | 21.2108898822 | 251 | 9e-66 |
| BAP05589.1 | CalA | BGC0000967 | Calyculin\_biosynthetic\_gene\_cluster | 34 | 20.9670865502 | 251 | 1.2e-65 |
| ABI91464.1 | beta-ketoacyl\_synthase | BGC0001094 | Enacyloxin\_biosynthetic\_gene\_cluster | 34 | 20.2763104429 | 251 | 1.2e-65 |
| AGZ15474.1 | putative\_type\_I\_polyketide\_synthase | BGC0001036 | Polyoxypeptin\_biosynthetic\_gene\_cluster | 27 | 33.6042259244 | 250 | 1.5e-65 |
| CAQ64687.1 | lasalocid\_modular\_polyketide\_synthase | BGC0000087 | Lasalocid\_biosynthetic\_gene\_cluster | 26 | 33.2791548151 | 250 | 2e-65 |
| CAN93352.1 | polyketide\_synthase | BGC0000179 | Etnangien\_biosynthetic\_gene\_cluster | 34 | 22.0642015441 | 250 | 2e-65 |
| BAG85027.1 | putative\_polyketide\_synthase | BGC0000086 | Lasalocid\_biosynthetic\_gene\_cluster | 26 | 33.2791548151 | 250 | 2e-65 |
| ACR13065.1 | modular\_polyketide\_synthase,\_type\_I\_PKS | BGC0000185 | Tartrolon\_biosynthetic\_gene\_cluster | 33 | 21.6172287688 | 249 | 4.4e-65 |
| BAP05590.1 | CalB | BGC0000967 | Calyculin\_biosynthetic\_gene\_cluster | 32 | 24.1365298659 | 249 | 4.4e-65 |
| CCA89329.1 | trans-AT\_type\_I\_polyketide\_synthase | BGC0001111 | Rhizopodins\_biosynthetic\_gene\_cluster | 33 | 20.6013815522 | 249 | 4.4e-65 |
| CAL69894.1 | RhiF\_protein | BGC0001112 | Rhizoxins\_biosynthetic\_gene\_cluster | 33 | 20.8451848842 | 247 | 1.3e-64 |
| ABS74181.1 | BmyA | BGC0001090 | Bacillomycin\_biosynthetic\_gene\_cluster | 34 | 20.8045509955 | 247 | 1.7e-64 |
| BAB69698.1 | iturin\_A\_synthetase\_A | BGC0001098 | Iturin\_biosynthetic\_gene\_cluster | 34 | 20.8045509955 | 247 | 1.7e-64 |
| AAF08795.1 | MycA | BGC0001103 | Mycosubtilin\_biosynthetic\_gene\_cluster | 34 | 20.4388459976 | 247 | 2.2e-64 |
| CAJ57410.1 | polyketide\_synthase\_type\_I | BGC0000176 | Difficidin\_biosynthetic\_gene\_cluster | 34 | 20.9264526615 | 247 | 2.2e-64 |
| ALA62323.1 | methylphloroacetophenone\_synthase | BGC0001309 | Usnic\_acid\_biosynthetic\_gene\_cluster | 33 | 21.2515237708 | 246 | 2.9e-64 |
| AAZ77694.1 | ChlA2 | BGC0000036 | Chlorothricin\_biosynthetic\_gene\_cluster | 25 | 31.6944331573 | 245 | 4.9e-64 |
| ABC34832.1 | polyketide\_synthase | BGC0000186 | Thailandamide\_biosynthetic\_gene\_cluster | 34 | 20.6013815522 | 245 | 4.9e-64 |
| ABM63529.1 | BryX | BGC0000174 | Bryostatin\_biosynthetic\_gene\_cluster | 32 | 21.6578626575 | 245 | 4.9e-64 |
| ADH01484.1 | putative\_type-I\_PKS | BGC0001096 | FR901464\_biosynthetic\_gene\_cluster | 35 | 21.2515237708 | 245 | 4.9e-64 |
| CAG23978.1 | polyketide\_synthase\_type\_I | BGC0000176 | Difficidin\_biosynthetic\_gene\_cluster | 34 | 21.4140593255 | 245 | 4.9e-64 |
| AIC32692.1 | FR9C | BGC0001113 | Spliceostatin\_/\_FR901464\_biosynthetic\_gene\_cluster | 35 | 21.2515237708 | 245 | 4.9e-64 |
| ACY01392.1 | AT-less\_polyketide\_synthase | BGC0000177 | Dorrigocin\_/\_migrastatin\_biosynthetic\_gene\_cluster | 33 | 20.6826493295 | 245 | 6.4e-64 |
| AEZ53946.1 | polyketide\_synthase | BGC0000144 | Salinomycin\_biosynthetic\_gene\_cluster | 28 | 27.3059731816 | 245 | 6.4e-64 |
| CAL69889.1 | RhiB\_protein | BGC0001112 | Rhizoxins\_biosynthetic\_gene\_cluster | 34 | 21.4546932141 | 245 | 6.4e-64 |
| AFN27483.1 | pks\_BonD | BGC0000173 | Bongkrekic\_acid\_biosynthetic\_gene\_cluster | 33 | 21.2108898822 | 245 | 6.4e-64 |
| ADC45586.1 | modular\_polyketide\_synthase | BGC0000093 | Meilingmycin\_biosynthetic\_gene\_cluster | 29 | 27.9561154002 | 245 | 8.4e-64 |
| AFK83820.1 | PtzD | BGC0001107\_c2 | Patellazole\_biosynthetic\_gene\_cluster | 32 | 20.7639171069 | 244 | 1.1e-63 |
| ABM63537.1 | BryA | BGC0000174 | Bryostatin\_biosynthetic\_gene\_cluster | 32 | 22.0235676554 | 244 | 1.4e-63 |
| AFK83819.1 | PtzC | BGC0001107 | Patellazole\_biosynthetic\_gene\_cluster | 34 | 21.8610321008 | 244 | 1.4e-63 |
| CAN89634.1 | putative\_polyketide\_synthase | BGC0001070 | Kirromycin\_biosynthetic\_gene\_cluster | 32 | 20.6826493295 | 243 | 2.4e-63 |
| AEP40936.1 | polyketide\_synthase\_type\_I | BGC0000021 | Apoptolidin\_biosynthetic\_gene\_cluster | 27 | 35.0264120276 | 243 | 3.2e-63 |
| AAM12913.2 | MmpD | BGC0000182 | Mupirocin\_biosynthetic\_gene\_cluster | 34 | 21.2921576595 | 243 | 3.2e-63 |
| AEC04364.1 | polyketide\_synthase | BGC0000178 | Elansolid\_biosynthetic\_gene\_cluster | 33 | 21.6984965461 | 242 | 5.4e-63 |
| AGS06887.1 | mixed\_type\_I\_polyketide\_synthase/non-ribosomal\_peptide\_synthetase,\_PedF-like\_protein | BGC0001092\_c2 | Diaphorin\_biosynthetic\_gene\_cluster | 32 | 21.2108898822 | 241 | 1.2e-62 |
| AFK57216.1 | DidE | BGC0000985 | Didemnin\_biosynthetic\_gene\_cluster | 34 | 21.2515237708 | 240 | 2.1e-62 |
| CUI25743.1 | Polyketide\_synthase | BGC0001354 | U-68204\_biosynthetic\_gene\_cluster | 31 | 20.3169443316 | 240 | 2.7e-62 |
| CAN93350.1 | polyketide\_synthase | BGC0000179 | Etnangien\_biosynthetic\_gene\_cluster | 32 | 20.5607476636 | 239 | 3.5e-62 |
| AHD05679.1 | putative\_non-ribosomal\_peptide\_ligase/\_polyketide\_synthase\_hybrid | BGC0000402 | Paenilarvins\_biosynthetic\_gene\_cluster | 33 | 21.5359609915 | 238 | 6e-62 |
| AFX60341.1 | polyketide\_synthase | BGC0001032 | Oocydin\_A\_biosynthetic\_gene\_cluster | 32 | 22.1454693214 | 238 | 6e-62 |
| ALV82446.1 | putative\_type\_I\_polyketide\_synthase | BGC0001382 | Svaricin\_biosynthetic\_gene\_cluster | 32 | 20.7639171069 | 238 | 7.8e-62 |
| ABM63530.1 | BryD | BGC0000174 | Bryostatin\_biosynthetic\_gene\_cluster | 31 | 22.0235676554 | 237 | 1.3e-61 |
| AAN85522.1 | hybrid\_nonribosomal\_peptide\_synthetase\_/\_polyketide\_synthase | BGC0001101 | Leinamycin\_biosynthetic\_gene\_cluster | 32 | 22.551808208 | 237 | 1.7e-61 |
| ALJ49922.1 | TtmG | BGC0001236 | Thiotetroamide\_biosynthetic\_gene\_cluster | 32 | 20.4388459976 | 236 | 3e-61 |
| CCA89327.1 | trans-AT\_type\_I\_polyketide\_synthase | BGC0001111 | Rhizopodins\_biosynthetic\_gene\_cluster | 33 | 20.6013815522 | 236 | 3.9e-61 |
| BAP05595.1 | CalG | BGC0000967 | Calyculin\_biosynthetic\_gene\_cluster | 34 | 20.6420154409 | 235 | 5.1e-61 |
| EAU29529.1 | hypothetical\_protein | BGC0000682 | Terretonin\_biosynthetic\_gene\_cluster | 30 | 25.8837870784 | 234 | 1.1e-60 |
| AGN11880.1 | TstC | BGC0001114 | Thailanstatin\_biosynthetic\_gene\_cluster | 34 | 20.4388459976 | 233 | 1.9e-60 |
| AIJ04681.1 | polyketide\_synthase | BGC0001383 | Macrolactin\_biosynthetic\_gene\_cluster | 30 | 21.0889882162 | 233 | 3.3e-60 |
| CBW45692.1 | putative\_modular\_polyketide\_synthase\_(fragment) | BGC0000952\_c3 | Pristinamycin\_biosynthetic\_gene\_cluster | 33 | 20.5201137749 | 232 | 4.3e-60 |
| CAG23964.1 | polyketide\_synthase\_type\_I | BGC0000181 | Macrolactin\_biosynthetic\_gene\_cluster | 30 | 21.0889882162 | 232 | 5.6e-60 |
| AAY32966.1 | DszC | BGC0001093 | Disorazole\_biosynthetic\_gene\_cluster | 33 | 22.6330759854 | 231 | 9.6e-60 |
| AAP42873.1 | NanA11 | BGC0000105 | Nanchangmycin\_biosynthetic\_gene\_cluster | 26 | 33.3197887038 | 230 | 1.6e-59 |
| CAN93349.1 | polyketide\_synthase | BGC0000179 | Etnangien\_biosynthetic\_gene\_cluster | 33 | 21.5765948801 | 230 | 2.1e-59 |
| AFN27481.1 | pks\_BonB | BGC0000173 | Bongkrekic\_acid\_biosynthetic\_gene\_cluster | 33 | 21.0077204388 | 230 | 2.1e-59 |
| AFL48526.1 | laidlomycin\_polyketide\_synthase\_(module\_2) | BGC0000084 | Laidlomycin\_biosynthetic\_gene\_cluster | 27 | 27.5497765136 | 229 | 4.8e-59 |
| AAM12925.2 | MmpE | BGC0000182 | Mupirocin\_biosynthetic\_gene\_cluster | 32 | 20.7232832182 | 228 | 8.1e-59 |
| CAD15512.1 | probable\_polyketide\_synthase\_protein | BGC0001014 | Micacocidin\_biosynthetic\_gene\_cluster | 33 | 21.3327915482 | 228 | 1.1e-58 |
| ADH01485.1 | putative\_mixed\_polyketide\_synthase/non-ribosomal\_peptide\_synthetase | BGC0001096 | FR901464\_biosynthetic\_gene\_cluster | 33 | 20.6826493295 | 225 | 5.3e-58 |
| ADN68480.1 | SorE | BGC0000184 | Sorangicin\_biosynthetic\_gene\_cluster | 32 | 22.3080048761 | 225 | 9e-58 |
| AFX60318.1 | polyketide\_synthase | BGC0001031 | Oocydin\_A\_biosynthetic\_gene\_cluster | 31 | 22.1454693214 | 224 | 1.2e-57 |
| ABS90472.1 | PKS | BGC0001106 | Oxazolomycin\_biosynthetic\_gene\_cluster | 31 | 20.9264526615 | 223 | 2e-57 |
| ABC84457.1 | NigAII | BGC0000114 | Nigericin\_biosynthetic\_gene\_cluster | 25 | 31.8163348232 | 223 | 2.6e-57 |
| ABC84470.1 | NIGAVIII | BGC0000114 | Nigericin\_biosynthetic\_gene\_cluster | 25 | 33.6042259244 | 221 | 9.9e-57 |
| AAP42856.1 | NanA2 | BGC0000105 | Nanchangmycin\_biosynthetic\_gene\_cluster | 26 | 31.9788703779 | 221 | 1.3e-56 |
| ACR50791.1 | putative\_polyketide\_synthase | BGC0000163 | Tetronasin\_biosynthetic\_gene\_cluster | 30 | 22.2267370987 | 220 | 2.2e-56 |
| ABP57746.1 | DepB | BGC0000993 | FK228\_biosynthetic\_gene\_cluster | 31 | 21.7391304348 | 219 | 4.9e-56 |
| ABC35027.1 | JamP | BGC0000961 | Bactobolin\_biosynthetic\_gene\_cluster | 32 | 21.2515237708 | 218 | 1.1e-55 |
| ABC38737.1 | polyketide\_synthase | BGC0000964 | Burkholdac\_biosynthetic\_gene\_cluster | 29 | 28.0780170662 | 217 | 1.9e-55 |
| BAP05596.1 | CalH | BGC0000967 | Calyculin\_biosynthetic\_gene\_cluster | 30 | 20.8858187729 | 217 | 2.4e-55 |
| CAJ76289.1 | putative\_hybrid\_non-ribosomal\_peptide-polyketide\_synthetase | BGC0000972 | Colibactin\_biosynthetic\_gene\_cluster | 29 | 26.4932954084 | 216 | 3.2e-55 |
| AGN74893.1 | nonribosomal\_peptide\_synthetase/polyketide\_synthase\_hybrid\_protein | BGC0000459 | Griseoviridin\_/\_viridogrisein\_biosynthetic\_gene\_cluster | 31 | 22.79561154 | 215 | 5.4e-55 |
| AFK57218.1 | DidG | BGC0000985 | Didemnin\_biosynthetic\_gene\_cluster | 31 | 23.40511987 | 215 | 7.1e-55 |
| AAP42859.1 | NanA5 | BGC0000105 | Nanchangmycin\_biosynthetic\_gene\_cluster | 27 | 32.2226737099 | 215 | 9.3e-55 |
| CBK62718.1 |  | BGC0001115 | Thiomarinol\_biosynthetic\_gene\_cluster | 31 | 20.8858187729 | 212 | 7.9e-54 |
| AFR69332.1 | polyketide\_synthase\_SpiB | BGC0001045 | Spiruchostatin\_biosynthetic\_gene\_cluster | 30 | 20.6013815522 | 206 | 4.3e-52 |
| AAL01060.1 | omega-3\_polyunsaturated\_fatty\_acid\_synthase\_PfaA | BGC0000865 | Eicoseicosapentaenoic\_acid\_biosynthetic\_gene\_cluster | 28 | 28.8500609508 | 205 | 5.6e-52 |
| AGJ76601.1 | HglE | BGC0000869 | Heterocyst\_glycolipids\_biosynthetic\_gene\_cluster | 27 | 29.6627387241 | 201 | 1.1e-50 |
| ACO79122.1 | Type\_I\_fatty\_acid\_synthase\_ArsA | BGC0000284 | Phenolic\_lipids\_biosynthetic\_gene\_cluster | 26 | 30.5973181633 | 197 | 1.5e-49 |
| ADO69378.1 | beta-ketoacyl\_synthase-like\_protein,\_Acyl\_transferase\_subunit | BGC0000044 | Dawenol\_biosynthetic\_gene\_cluster | 28 | 28.2405526209 | 195 | 1e-48 |
| CBK62729.1 |  | BGC0001115 | Thiomarinol\_biosynthetic\_gene\_cluster | 30 | 21.3327915482 | 194 | 1.3e-48 |
| AMK92560.1 | enediyne\_polyketide\_synthase | BGC0001377 | Uncialamycin\_biosynthetic\_gene\_cluster | 29 | 26.9402681837 | 193 | 2.2e-48 |
| CAG23960.2 | hybrid\_NRPS/PKS | BGC0001089 | Bacillaene\_biosynthetic\_gene\_cluster | 29 | 21.4140593255 | 191 | 1.1e-47 |
| BAF02922.1 | type\_I\_polyketide\_synthase | BGC0000073 | Halstoctacosanolide\_biosynthetic\_gene\_cluster | 26 | 27.7935798456 | 190 | 1.9e-47 |
| AGC09472.1 | crotonyl-CoA\_reductase/alcohol\_dehydrogenase | BGC0001183 | Lobophorin\_biosynthetic\_gene\_cluster | 26 | 24.0146281999 | 189 | 4.2e-47 |
| BAF02924.1 | type\_I\_polyketide\_synthase | BGC0000073 | Halstoctacosanolide\_biosynthetic\_gene\_cluster | 26 | 27.7935798456 | 189 | 5.5e-47 |
| AAM78012.1 | warhead-forming\_iterative\_polyketide\_synthase | BGC0000112 | Neocarzinostatin\_biosynthetic\_gene\_cluster | 27 | 29.5408370581 | 186 | 3.5e-46 |
| AAB81123.1 | unknown | BGC0000862 | Eicosapentaenoic\_acid\_biosynthetic\_gene\_cluster | 25 | 37.0581064608 | 186 | 4.6e-46 |
| BAA89382.2 |  | BGC0000864 | Eicosapentaenoic\_acid-like\_compound\_biosynthetic\_gene\_cluster | 27 | 29.0125965055 | 181 | 1.1e-44 |
| ABF00127.1 | polyunsaturated\_fatty\_acid\_synthase | BGC0000863 | Eicosapentaenoic\_acid\_biosynthetic\_gene\_cluster | 27 | 29.8252742787 | 172 | 5.3e-42 |
| ACI12948.1 | PfaA | BGC0000861 | Eicosapentaenoic\_acid\_biosynthetic\_gene\_cluster | 26 | 20.1950426656 | 146 | 3.1e-34 |
